# Plant-Derived Compounds as Antibiotic Adjuvants Against Drug-Resistant ESKAPE Pathogens: Mechanisms of Action, Synergistic Strategies, and Translational Challenges

**DOI:** 10.3390/antibiotics15080761

**Published:** 2026-08-07

**Authors:** Stefano Ruga, Elisa Matarese, Fabio Castagna, Clementina Sansone, Canio Martinelli, Giulio Mazzarotti, Andrea Russo, Annamaria Medugno, Shendi Shani, Antonio Giordano, Roberto Bava, Giovanna Liguori

**Affiliations:** 1 Department of Health Sciences, Magna Graecia University of Catanzaro, 88100 Catanzaro, Italy; stefano.ruga@studenti.unicz.it (S.R.); fabiocastagna@unicz.it (F.C.); 2 Vita-Salute San Raffaele University, 20132 Milan, Italy; e.matarese@studenti.unisr.it; 3 Department of Eco-Sustainable Marine Biotechnologies, Anton Dohrn Zoological Station, Molosiglio Marina di Acton, 80133 Naples, Italy; clementina.sansone@szn.it; 4 Sbarro Institute for Cancer Research and Molecular Medicine, Center for Biotechnology, Department of Biology, College of Science and Technology, Temple University, Philadelphia, PA 19122, USA; canio.martinelli@shro.org (C.M.); giulio.mazzarotti@hotmail.it (G.M.); russo.andrea24396@gmail.com (A.R.); medugnomedu@gmail.com (A.M.); shani95@hotmail.it (S.S.); antonio.giordano@temple.edu (A.G.); giovanna.liguori@aslfg.it (G.L.); 5 Sbarro Health Research Organization ETS, 10060 Candiolo, Italy; 6 Department of Medical Biotechnologies, University of Siena, 53100 Siena, Italy; 7 Local Health Authority of Foggia (ASL Foggia), 71121 Foggia, Italy

**Keywords:** natural products, phytodrugs, anti-infective agents, antivirulence strategies, antimicrobial activity, human pathogens, quorum sensing inhibition, fractional inhibitory concentration (FIC), nanocarriers

## Abstract

**Background/Objectives**: Antimicrobial resistance among ESKAPE (*Enterococcus faecium*, *Staphylococcus aureus*, *Klebsiella pneumoniae*, *Acinetobacter baumannii*, *Pseudomonas aeruginosa*, and *Enterobacter* species) pathogens has become a major global health threat, significantly reducing the effectiveness of conventional antibiotics. Plant-derived compounds have emerged as promising antibiotic adjuvants capable of restoring antibiotic activity through multiple resistance-modulating mechanisms. This review aims to critically evaluate the current evidence regarding phytochemicals that enhance antibiotic efficacy against drug-resistant ESKAPE pathogens. **Methods**: Relevant studies investigating plant-derived compounds with antibiotic adjuvant activity against ESKAPE pathogens were identified and critically analyzed. Particular attention was given to mechanisms of action, synergistic interactions with conventional antibiotics, structure–activity relationships, and translational evidence from in vivo studies. **Results**: Numerous phytochemicals have demonstrated the ability to potentiate antibiotic activity through efflux pump inhibition, biofilm disruption, membrane permeabilization, and quorum sensing interference. For instance, the flavonoid quercetin inhibits the NorA efflux pump in *Staphylococcus aureus*, restoring ciprofloxacin susceptibility, while the sulfur-containing compound ajoene from garlic disrupts quorum sensing in *Pseudomonas aeruginosa*, sensitizing biofilms to tobramycin. Although several compounds exhibit promising synergistic effects in vitro, significant barriers remain regarding bioavailability, standardization, pharmacokinetics, and clinical translation. **Conclusions**: Plant-derived antibiotic adjuvants represent a promising strategy to combat multidrug-resistant ESKAPE pathogens. However, greater emphasis on translational research, standardized methodologies, and clinically relevant experimental models is required to facilitate their development toward therapeutic applications.

## 1. Introduction

Antimicrobial resistance (AMR) represents one of the most pressing global health challenges of the 21st century, threatening to undermine decades of progress in modern medicine [1,2]. The World Health Organization (WHO) has identified a priority list of antibiotic-resistant bacteria, among which the so-called ESKAPE pathogens—*Enterococcus faecium*, *Staphylococcus aureus*, *Klebsiella pneumoniae*, *Acinetobacter baumannii*, *Pseudomonas aeruginosa*, and *Enterobacter* species—pose the greatest threat to human health due to their ability to evade conventional antibiotic therapies and cause severe healthcare-associated infections [3,4]. Recent epidemiological data from Southern Italy and other European regions confirm that the prevalence of multidrug-resistant (MDR) ESKAPE strains continues to rise, significantly complicating clinical management and increasing mortality rates [5]. This alarming trend is not confined to high-income settings; cross-sectional studies conducted in hospitals in low- and middle-income countries such as Nepal have documented similarly high rates of MDR ESKAPE pathogens, underscoring the global nature of this crisis [6]. Similarly, retrospective epidemiological studies conducted at university hospitals in Italy have documented the clinical impact and resistance trends of ESKAPE pathogens, reinforcing the urgent need for new therapeutic strategies [7]. It has been estimated that by 2050, antimicrobial-resistant infections could cause 10 million deaths annually worldwide, with devastating economic and social consequences [8]. A recent study demonstrated that ESKAPE pathogens can develop clinically relevant resistance to antibiotics currently in development within only 60 days of laboratory exposure, and that resistance mutations are already present in natural bacterial populations, underscoring the urgency of the AMR crisis [9].

The ESKAPE pathogens encompass both Gram-positive and Gram-negative bacteria, each presenting distinct structural features that fundamentally influence their susceptibility or resistance to antibiotics [3,4]. Among the Gram-positive members, *Enterococcus faecium* and *Staphylococcus aureus* possess a thick peptidoglycan layer (20–80 nm) that serves as the primary structural barrier but lacks the additional outer membrane characteristic of Gram-negative species. This thick peptidoglycan layer is essential for maintaining cell shape and resisting osmotic pressure, and its synthesis is the target of critically important antibiotics such as vancomycin and the β-lactams [4,10]. However, *S. aureus* has evolved resistance to methicillin through the acquisition of the *mecA* gene, which encodes an altered penicillin-binding protein (PBP2a) with reduced affinity for β-lactams, rendering this entire antibiotic class ineffective [4]. *E. faecium* has similarly acquired vancomycin resistance through the *van* gene cluster, which alters the peptidoglycan precursor from D-Ala-D-Ala to D-Ala-D-Lac, dramatically reducing vancomycin binding affinity [10,11].

The Gram-negative members of the ESKAPE group, *Klebsiella pneumoniae*, *Acinetobacter baumannii*, *Pseudomonas aeruginosa*, and *Enterobacter* species, present a more formidable challenge due to their unique cell envelope architecture. In addition to a thinner peptidoglycan layer (5–10 nm), these bacteria possess an outer membrane composed of lipopolysaccharides (LPS), phospholipids, and proteins, which functions as a highly selective permeability barrier [12,13]. The outer membrane restricts the passive diffusion of hydrophobic antibiotics (e.g., macrolides, rifampicin) and many hydrophilic agents that exceed the size exclusion limit of porin channels, contributing to intrinsic resistance [12,13]. Furthermore, the periplasmic space between the inner and outer membranes houses β-lactamase enzymes that can hydrolyze β-lactam antibiotics before they reach their periplasmic targets [14]. *P. aeruginosa* is particularly problematic due to its relatively low outer membrane permeability (12–100 times less permeable than that of *E. coli*) and its ability to upregulate efflux pumps of the Resistance-Nodulation-Division (RND) family, such as MexAB-OprM, which actively extrude a broad range of antibiotics [12,13]. *A. baumannii* and *K. pneumoniae* have similarly demonstrated a remarkable capacity to acquire resistance through horizontal gene transfer, including carbapenemases (e.g., KPC, NDM, OXA-type enzymes) that hydrolyze last-resort carbapenem antibiotics [4,14]. These structural and adaptive features, summarized in Table 1, explain why Gram-negative ESKAPE pathogens are among the most difficult to treat and underscore the need for adjuvant strategies that can overcome or bypass these intrinsic and acquired resistance barriers.

The economic dimension of the AMR crisis further compounds the clinical challenge. The estimated cost of developing a new antibiotic from discovery to market approval has been estimated at approximately $1.5 billion, yet the return on investment is often negative due to the relatively short treatment duration, the inevitable emergence of resistance, and the imperative of antibiotic stewardship that restricts the use of new agents to preserve their efficacy [16,17]. This unfavorable economic equation has led many major pharmaceutical companies to abandon antibiotic research and development over the past two decades, resulting in a dangerously thin pipeline of novel antimicrobial agents [17]. In this context, antibiotic adjuvants derived from natural products may offer a more economically attractive alternative: by restoring the activity of existing, off-patent antibiotics, the development costs could potentially be lower than those for entirely new chemical entities, although this hypothesis remains to be demonstrated in practice [18,19].

The traditional paradigm of antibiotic development, discovering new molecules that directly kill bacteria or inhibit their growth, has proven increasingly unsustainable. The rapid emergence of resistance mechanisms, including enzymatic drug inactivation, target modification, reduced membrane permeability, and active efflux of antibiotics, has outpaced the discovery of novel therapeutic agents [20,21]. Consequently, there is an urgent need for innovative strategies that can restore the efficacy of existing antibiotics rather than relying solely on the development of new ones [18]. In this context, the concept of “antibiotic adjuvants” has gained considerable attention. Unlike conventional antibiotics, adjuvants do not necessarily possess direct antimicrobial activity; instead, they modulate bacterial physiology or resistance mechanisms, thereby re-sensitizing resistant pathogens to conventional antibiotics [19,22]. Dhanda et al. (2023) further classified antibiotic adjuvants into three broad categories: Class I direct resistance breakers (e.g., enzyme inhibitors, efflux pump inhibitors), Class Ib indirect resistance breakers (e.g., membrane-targeting compounds, teichoic acid biosynthesis inhibitors), and Class II host-modulating agents that enhance innate immune responses. This classification provides a useful framework for understanding the diverse mechanisms by which plant-derived compounds can potentiate antibiotic activity [19]. As comprehensively reviewed by Liu et al. (2019), antibiotic adjuvants can be categorized based on their modes of action: inhibitors of resistance enzymes (e.g., β-lactamase inhibitors), agents that enhance intracellular antibiotic accumulation by inhibiting efflux pumps or permeabilizing the outer membrane, compounds with complementary bactericidal mechanisms, inhibitors of signaling and regulatory pathways, and agents that enhance host defense responses. The authors emphasized that Gram-negative bacteria present a particularly challenging target due to their outer membrane permeability barrier, making adjuvants that disrupt this barrier, such as the anti-protozoal drug pentamidine, which interacts with lipopolysaccharide to potentiate rifampicin and novobiocin against *A. baumannii*, especially valuable [23]. This conceptual framework is directly applicable to plant-derived adjuvants, which have been shown to act through several of these mechanisms, particularly efflux pump inhibition and membrane permeabilization [23].

Historically, plants have served as an invaluable source of bioactive compounds with diverse pharmacological properties, including antimicrobial, anti-inflammatory, and immunomodulatory activities [24,25]. The potential of plants as sources of bacterial resistance modulators and anti-infective agents has been recognized for decades, with early reviews highlighting the chemical diversity and therapeutic promise of phytochemicals in overcoming antibiotic resistance [26]. The use of plant-derived extracts and phytochemicals in traditional medicine systems worldwide has provided a rich repository of knowledge that modern science is increasingly leveraging to address contemporary health challenges [27,28]. In recent years, research has shifted from the search for novel plant-derived antibiotics toward the identification of phytochemicals that can function as antibiotic adjuvants, capable of inhibiting bacterial efflux pumps, disrupting biofilm formation, interfering with quorum sensing (QS) signaling systems, and permeabilizing bacterial membranes [29,30]. A comprehensive review by Bhatia et al. (2021) catalogued 100 medicinal plants from 12 countries across Asia, Africa, Europe, and the Americas with documented antimicrobial activity against ESKAPE pathogens, with India alone contributing 35 species. Notably, several plants, including *Martynia annua*, *Rauwolfia serpentina*, *Piper betle*, and *Ehretia microphylla*, exhibited broad-spectrum activity against multiple ESKAPE members, highlighting the vast ethnopharmacological reservoir available for the discovery of novel antibiotic adjuvants [31]. These mechanisms not only enhance the efficacy of conventional antibiotics but also reduce the selective pressure that drives the emergence of resistance, representing a paradigm shift from bactericidal to anti-virulence strategies [32].

Despite promising in vitro evidence demonstrating the ability of plant-derived compounds to potentiate antibiotic activity against MDR ESKAPE pathogens, significant barriers remain in translating these findings into clinical applications. Issues related to pharmacokinetics, bioavailability, standardization of extracts, cytotoxicity, and the lack of robust in vivo models have hampered the progression of phytochemical adjuvants from the laboratory to the bedside [33,34]. Moreover, the complexity of plant extracts, which often contain multiple bioactive compounds that may act synergistically or antagonistically, complicates the identification of specific mechanisms of action and the optimization of therapeutic formulations [35,36]. In this context, it has been authoritatively observed that the inherent complexity of plant extracts, while posing formidable challenges for standardization and quality control, simultaneously represents a distinct advantage over single chemical entity medicines, as the polypharmacological action of multiple components can enhance activity through synergistic interactions and reduce the likelihood of resistance development [29].

This review aims to critically evaluate the current evidence regarding plant-derived compounds that function as antibiotic adjuvants against drug-resistant ESKAPE pathogens. We will examine the principal mechanisms of action through which phytochemicals enhance antibiotic efficacy, including efflux pump inhibition, biofilm disruption, quorum sensing interference, and membrane permeabilization. Furthermore, we will discuss synergistic strategies combining phytochemicals with conventional antibiotics, highlighting methodological approaches for evaluating synergy and providing examples of successful re-sensitization of ESKAPE pathogens to first-line therapies. Finally, we will address the translational challenges that must be overcome to advance plant-derived adjuvants toward clinical applications, including pharmacokinetic limitations, toxicity concerns, and the potential of nanotechnology and advanced delivery systems to bridge the gap between in vitro promise and in vivo efficacy. By synthesizing the most recent findings and identifying key research gaps, this review seeks to provide a comprehensive framework for future investigations into plant-derived antibiotic adjuvants as a viable strategy to combat the growing crisis of antimicrobial resistance.

## 2. Mechanisms of Action of Plant-Derived Adjuvants Against ESKAPE Pathogens

**Figure 1:**
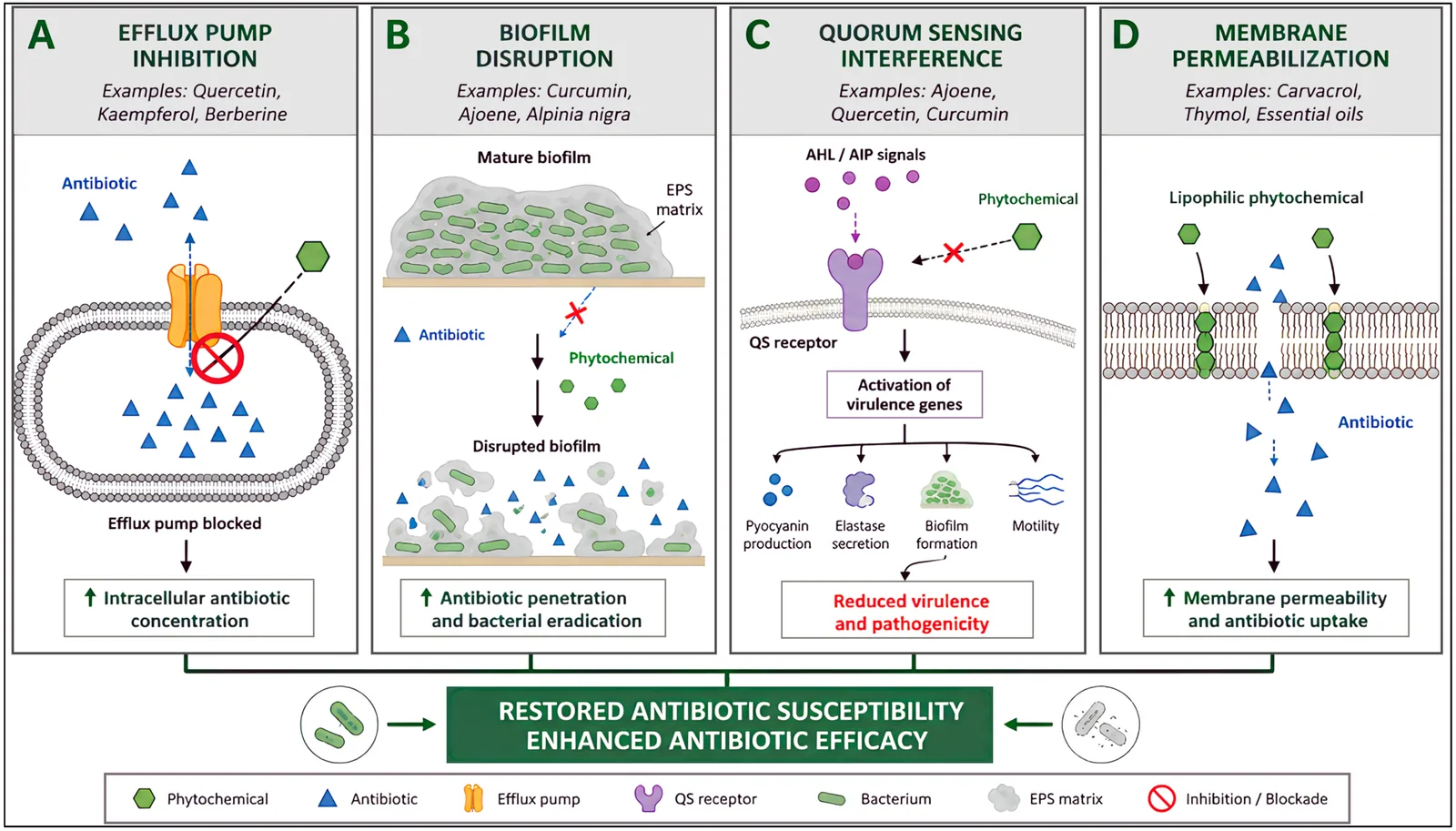
Mechanisms by which plant-derived phytochemicals enhance antibiotic efficacy against multidrug-resistant ESKAPE (*Enterococcus faecium*, *Staphylococcus aureus*, *Klebsiella pneumoniae*, *Acinetobacter baumannii*, *Pseudomonas aeruginosa*, and *Enterobacter* species) pathogens. Plant-derived adjuvants potentiate conventional antibiotics through four major mechanisms: (**A**) inhibition of bacterial efflux pumps, leading to increased intracellular antibiotic accumulation; (**B**) disruption of the extracellular polymeric substance (EPS) matrix and biofilm architecture, exposing embedded bacterial cells to antibiotic penetration; (**C**) interference with quorum sensing (QS) signaling, reducing the expression of virulence factors and impairing biofilm maturation; and (**D**) permeabilization of the bacterial cell envelope, facilitating antibiotic uptake. Although illustrated as distinct mechanisms, many phytochemicals act through multiple complementary pathways, collectively restoring antibiotic susceptibility and enhancing antibacterial efficacy against multidrug-resistant ESKAPE pathogens. Abbreviations: AHL: N-acyl homoserine lactone; AIP: Autoinducing peptide; EPS: Extracellular polymeric substance; QS: Quorum sensing.

The capacity of plant-derived compounds to restore antibiotic efficacy against multidrug-resistant ESKAPE pathogens relies on a diverse array of mechanisms that target bacterial resistance and virulence without necessarily exerting direct bactericidal pressure (Figure 1). Unlike conventional antibiotics, which typically act on essential cellular processes such as cell wall synthesis, protein synthesis, or nucleic acid replication, antibiotic adjuvants modulate non-essential but clinically relevant phenotypes that enable pathogens to survive antibiotic exposure [18,19]. The principal mechanisms through which phytochemicals potentiate antibiotic activity include the inhibition of efflux pumps, disruption of biofilm formation, interference with quorum sensing systems, and permeabilization of bacterial membranes [30,37,38,39]. Understanding these mechanisms is crucial for the rational development of phytochemical-based adjuvant therapies and to overcome the challenges that currently limit their clinical application.

### 2.1. Inhibition of Efflux Pumps (EPIs)

Efflux pumps represent one of the most clinically significant resistance mechanisms employed by ESKAPE pathogens, actively extruding a broad spectrum of structurally unrelated antibiotics from the bacterial cell and thereby reducing their intracellular concentrations to sub-therapeutic levels [1,40]. The fundamental architecture and functional classification of bacterial efflux systems, including the major facilitator superfamily (MFS), the resistance-nodulation-division (RND) family, and the multidrug and toxic compound extrusion (MATE) family, have been comprehensively reviewed, providing a structural framework for the rational development of efflux pump inhibitors [41]. The rationale for investigating plants as a source of EPIs is supported by a sound ecological logic: since plants coexist with soil bacteria, including plant pathogens, they have likely evolved secondary metabolites not only with direct antimicrobial activity but also with the capacity to inhibit bacterial resistance mechanisms such as efflux pumps, as part of their chemical defense strategy [29]. In Gram-negative ESKAPE pathogens such as *Pseudomonas aeruginosa*, *Acinetobacter baumannii*, and *Klebsiella pneumoniae*, the Resistance-Nodulation-Division (RND) superfamily pumps, including AcrAB-TolC and MexAB-OprM, are particularly problematic due to their broad substrate specificity and constitutive overexpression in multidrug-resistant clinical isolates [12,42]. In Gram-positive pathogens such as *Staphylococcus aureus*, the Major Facilitator Superfamily (MFS) pump NorA and the Multidrug and Toxic Compound Extrusion (MATE) family contribute significantly to fluoroquinolone and other antibiotic resistance [43,44].

The search for efflux pump inhibitors (EPIs) from natural sources has yielded numerous phytochemicals capable of blocking these transport proteins, thereby restoring the intracellular accumulation and antibacterial activity of co-administered antibiotics [29,45]. Pioneering work in this area led to the discovery of plant-derived compounds that specifically inhibit the NorA efflux pump in *S. aureus*, demonstrating the feasibility of targeting efflux-mediated resistance with natural products [46]. Table 2 summarizes representative plant-derived EPIs and their target efflux pumps in ESKAPE pathogens, illustrating the chemical diversity and broad-spectrum potential of these compounds.

Flavonoids constitute one of the most extensively studied classes of plant-derived EPIs. Waditzer and Bucar (2021) provided a comprehensive review of flavonoids as inhibitors of bacterial efflux pumps, demonstrating that compounds such as quercetin (abundant in apples, onions, and teas, as well as in medicinal plants like *Ginkgo biloba* and *Hypericum perforatum* [52]), kaempferol, and apigenin can effectively inhibit NorA-mediated efflux in *S. aureus* and potentiate the activity of fluoroquinolones. The structure–activity relationships of these compounds indicate that the presence of specific hydroxyl groups and the degree of lipophilicity are critical determinants of EPI potency [15,49].

Terpenes represent another important class of EPIs. A systematic review by Dias et al. (2022) evaluated the efflux pump inhibitory potential of terpenes, highlighting the ability of compounds such as carvacrol, thymol, and α-pinene to inhibit RND efflux pumps in Gram-negative bacteria. These lipophilic molecules are thought to interact with the hydrophobic domains of efflux pump proteins, potentially disrupting pump assembly or function [48,53]. The essential oil components from *Rosmarinus officinalis* have also demonstrated resistance-modifying activity, with carnosic acid and carnosol identified as compounds capable of inhibiting efflux pumps in *S. aureus* [47]. At a concentration of 10 μg/mL, carnosic acid caused an 8-fold potentiation of erythromycin activity against the MsrA effluxing strain RN4220, while carnosol produced a 4-fold potentiation of tetracycline against the TetK-expressing strain XU212. Interestingly, carnosic acid inhibited ethidium bromide efflux from the NorA-overexpressing strain SA-1199B with an IC50 of 50 μM, yet did not potentiate norfloxacin activity against this strain, suggesting inhibition of an alternative, as yet uncharacterized pump rather than NorA itself [47]. This observation aligns with the broader concept that the efflux landscape in *S. aureus* is more complex than previously appreciated, encompassing multiple pumps with overlapping substrate specificities [47,54].

Furthermore, several plant-derived compounds have been shown to act through indirect mechanisms of efflux pump inhibition. Michalet et al. (2007) identified N-caffeoylphenalkylamide derivatives as bacterial efflux pump inhibitors. These compounds, exemplified by *trans*-N-caffeoyltyramine and *trans*-N-feruloyltyramine, were isolated from *Polygonum hydropiper* and are also found in various plants including *Solanum melongena* (eggplant) and *Capsicum annuum* (bell pepper). They demonstrated the ability to reduce the expression of efflux pump genes rather than directly blocking pump activity, representing an attractive transcriptional-level strategy for reducing efflux-mediated resistance [50]. Omosa et al. (2016) screened a panel of 48 compounds from Kenyan plants and identified several with potent activity against multidrug-resistant phenotypes expressing active efflux pumps, further emphasizing the potential of plant biodiversity as a source of novel EPIs. Earlier work by Kuete et al. (2011) had similarly demonstrated that the naphthoquinone plumbagin, a compound with broad-spectrum activity against both Gram-positive and Gram-negative bacteria, showed a dramatic increase in potency when combined with PAβN, with MIC values decreasing from 16 to 32 mg/L to as low as 0.12–4 mg/L against MDR strains of *E. coli*, *E. aerogenes*, *K. pneumoniae*, and *P. aeruginosa*. The authors identified a common pharmacophore shared by the most active compounds, plumbagin, 4-hydroxylonchocarpin, laurentixanthone B, and the coumarin MAB3, confirming that specific structural features govern both antibacterial activity and susceptibility to efflux-mediated resistance [54]. Structure–activity relationship (SAR) studies of plant-derived benzoquinones have further revealed that antibacterial activity against MDR phenotypes increases progressively with increasing alkyl side chain length, with optimal activity achieved at a chain length of seven carbon atoms [51]. Similarly, SAR analysis of chalcones indicated that specific substitution patterns on ring B, namely a hydroxyl group at C3′ together with a methoxy group and a second hydroxyl group in meta orientation, are required for activity against MRSA [51]. These findings provide a rational basis for the medicinal chemistry optimization of plant-derived EPIs. Notably, the antibacterial activity of several compounds, particularly the anthraquinone emodin, was substantially enhanced (by >4–64 fold) when combined with the efflux pump inhibitor phenylalanine arginine β-naphthylamide (PAβN) against MDR Gram-negative strains of *E. coli* and *K. pneumoniae*, confirming that these phytochemicals are substrates of bacterial efflux pumps and underscoring the rationale for their development as EPIs or as companions to EPIs [51].

Despite the substantial body of in vitro evidence supporting the efficacy of plant-derived EPIs, several challenges remain. Many EPIs demonstrate potent activity in vitro but fail to translate to in vivo efficacy due to pharmacokinetic limitations and toxicity at concentrations required for pump inhibition [18,44]. Additionally, the substrate polyspecificity of RND pumps means that multiple EPIs may be required to achieve clinically meaningful sensitization, as different substrates may utilize distinct binding sites within the pump channel [40,55]. Furthermore, studies using PAβN in AcrAB-deleted strains of *E. coli* have revealed that additional, PAβN-sensitive efflux systems beyond the well-characterized AcrAB-TolC pump contribute to resistance against natural products, suggesting that the efflux landscape in MDR Gram-negative bacteria is more complex than previously appreciated and may require multi-target EPI strategies [54]. Taken together, the body of evidence on plant-derived EPIs demonstrates that while considerable progress has been made in identifying and characterizing these compounds in vitro, their translational value remains contingent upon overcoming pharmacokinetic limitations and target specificity issues.

### 2.2. Disruption of Biofilm Formation and Eradication

Biofilm formation represents a critical virulence mechanism in ESKAPE pathogens that significantly contributes to antimicrobial tolerance and treatment failure in clinical settings [56,57]. Biofilms are structured communities of bacterial cells embedded within a self-produced extracellular polymeric substance (EPS) matrix composed of polysaccharides, proteins, extracellular DNA, and lipids, which acts as a physical barrier to antibiotic penetration while also creating microenvironments conducive to the emergence of persister cells [58,59]. *P. aeruginosa* and *S. aureus* are particularly notorious for their ability to form robust biofilms on medical devices and in chronic wound infections, with biofilm-embedded cells exhibiting up to 1000-fold higher tolerance to antibiotics compared to their planktonic counterparts [60,61].

Plant-derived compounds have demonstrated considerable potential as anti-biofilm agents, capable of both inhibiting biofilm formation and eradicating pre-formed biofilms [62,63]. The mechanisms underlying these activities are multifaceted and include interference with initial surface attachment, inhibition of EPS matrix synthesis, disruption of intercellular signaling within the biofilm, and direct dispersal of established biofilms [64,65]. Uc-Cachón et al. (2021) investigated the antibacterial and antibiofilm activities of Mayan medicinal plants against methicillin-susceptible and methicillin-resistant strains of *S. aureus*, demonstrating that several plant extracts not only inhibited biofilm formation but also eradicated pre-formed biofilms at sub-inhibitory concentrations, suggesting the presence of specific anti-biofilm phytochemicals distinct from those responsible for antibacterial activity. Among the 15 Mayan plant species screened, the methanolic leaf extract of *Matayba oppositifolia* exhibited the most potent antibiofilm activity (IC50 = 10.4 μg/mL), while the methanolic bark extracts of *M. oppositifolia*, *Clusia flava*, and *Gymnopodium floribundum* displayed strong bactericidal effects against both MSSA and MRSA (MIC 250 μg/mL, MBC/MIC = 1). Notably, some aqueous extracts, including those of *Krugiodendron ferreum* bark and *M. oppositifolia* leaf, were more active against resistant strains (MRSA and MDRSA) than against susceptible ones, suggesting alternative modes of action that may specifically target resistance mechanisms. Phytochemical analysis of the most active extracts revealed terpenoids (72.7%), tannins (54.5%), and flavonoids (45.5%) as the predominant compound classes, consistent with the known membrane-disrupting and protein-denaturing properties of these phytochemicals [66].

Other well-characterized polyphenols have also shown significant antibiofilm potential against ESKAPE pathogens. Epigallocatechin gallate (EGCG), the major catechin in green tea (*Camellia sinensis*), has demonstrated the ability to inhibit biofilm formation by ocular staphylococcal isolates at sub-inhibitory concentrations [67] and to suppress quorum sensing and biofilm development in *P. aeruginosa* through interference with AHL-mediated signaling pathways [68]. Similarly, resveratrol, a stilbenoid found in grapes and red wine, has been reported to inhibit *Staphylococcus aureus* biofilm formation by reducing polysaccharide intercellular adhesin (PIA), extracellular DNA (eDNA) release, and reactive oxygen species (ROS) production, thereby enhancing the susceptibility of biofilm-embedded MRSA cells to conventional antibiotics [69]. These findings highlight the potential of dietary polyphenols as accessible and relatively non-toxic anti-biofilm adjuvants.

The anti-biofilm properties of flavonoids have been extensively documented. Quercetin, a flavonol widely distributed in fruits and vegetables, has demonstrated potent activity against biofilms formed by multiple ESKAPE pathogens [70,71]. Gupta et al. (2023) reported that a standardized methanolic leaf extract of *Alpinia nigra*, rich in flavonoids and phenolic compounds, effectively inhibited biofilm formation in *P. aeruginosa* PAO1 through a mechanism involving the suppression of quorum sensing-regulated virulence factors [72]. Similarly, Umamaheswari and SJ (2020) investigated the biofilm eradication potential of selected medicinal plants against methicillin-resistant *S. aureus* (MRSA), finding that certain extracts could disrupt established biofilms and restore vancomycin susceptibility in previously tolerant cells [73]. More recently, Barbarossa et al. (2025) evaluated the antibiofilm activity of commercial extracts from *Vitis vinifera* (grape seed), *Camellia sinensis* (green tea), *Olea europaea* (olive), *Quercus robur* (oak), and *Coffea arabica* (coffee) against both ATCC and clinical *S. aureus* strains. Notably, *Q. robur* extract, rich in tannins (75.1% polyphenols), exhibited the most potent antibacterial activity with MIC values as low as 0.2 mg/mL against clinical isolates and 0.4 mg/mL against MRSA ATCC 43300, while *C. arabica* extract, containing 25.2% caffeoylquinic acids, achieved up to 91.4% inhibition of biofilm formation at 128 mg/mL. The antibiofilm mechanisms identified included quorum sensing interference, disruption of exopolysaccharide synthesis, and membrane permeabilization, further validating the multi-target potential of plant-derived compounds [61].

Essential oils and their constituents have also emerged as promising anti-biofilm agents. Panda et al. (2022) reviewed recent advances in combating ESKAPE pathogens with special reference to essential oils, highlighting the capacity of these complex mixtures to penetrate the biofilm matrix and disrupt cellular membranes of embedded cells. The volatile nature and lipophilic character of essential oil components facilitate their diffusion through the EPS matrix, allowing them to reach and act upon cells deep within the biofilm structure [74,75]. Aati et al. (2025) investigated the antimicrobial and anti-biofilm activities of medicinal plant-derived honey against ESKAPE pathogens, providing insights into β-lactamase inhibition through metabolomics and molecular modeling studies. Their findings demonstrated that plant-derived compounds present in honey could simultaneously target multiple resistance mechanisms, including biofilm formation and enzymatic antibiotic inactivation. Notably, aloe honey (HO1) exhibited the most pronounced activity, inhibiting *S. aureus* growth by 93.3% and reducing *S. aureus* biofilm formation by 89.26% and *Salmonella typhimurium* biofilm by 85.12%. LC-MS metabolomic profiling of this honey identified 27 metabolites, predominantly phenolic acid derivatives, flavonoids, and anthraquinones. Molecular docking studies further revealed that two of these compounds, gallic acid hexoside and rosmarinic acid, displayed consensus docking scores superior to the clinically approved β-lactamase inhibitor avibactam across 10 classes of β-lactamases, suggesting their potential as competitive inhibitors capable of restoring β-lactam antibiotic activity [76]. While the study by Aati et al. (2025) focused on aloe honey, it is noteworthy that Manuka honey, derived from *Leptospermum scoparium*, is one of the most extensively investigated honeys for its antimicrobial and antibiofilm properties against ESKAPE pathogens [77]. Its potent activity is largely attributed to the unique Manuka factor (UMF), primarily methylglyoxal (MGO), which has demonstrated the ability to disrupt biofilm architecture, inhibit quorum sensing, and restore antibiotic susceptibility in MRSA and *P. aeruginosa* [78,79]. A comparative study by Lu et al. (2019) further demonstrated that Manuka honey effectively eradicates established biofilms of ESKAPE pathogens at clinically achievable concentrations [80]. This suggests that different honeys may offer complementary mechanisms of action worthy of further comparative investigation.

The anti-biofilm compounds and extracts discussed in this section, along with their sources, target pathogens, and mechanisms of action, are summarized in Table 3.

The clinical relevance of anti-biofilm strategies is underscored by the fact that biofilm-associated infections are inherently difficult to treat and often require prolonged antibiotic therapy or surgical intervention [56,81]. Despite biofilms being associated with approximately two-thirds of all human infections, no antibiotics have been specifically approved for biofilm eradication to date, as conventional antibiotic discovery platforms have historically focused on planktonic bacteria rather than biofilm-embedded communities [56]. This critical gap underscores the urgent need for alternative approaches, including plant-derived compounds that can either prevent biofilm formation or potentiate the activity of conventional antibiotics against biofilm-embedded cells [56]. In the context of mycobacterial infections, Matlala et al. (2024) demonstrated that an acetone extract of *Artemisia afra* and its subfraction FC2 not only inhibited planktonic growth of *Mycobacterium smegmatis* (MIC 0.078–0.833 mg/mL) but also reduced mycobacterial cell adherence and early biofilm formation by more than 50%. However, mature 48-h biofilms proved resistant to treatment, highlighting the critical importance of early intervention in biofilm-associated infections. Molecular docking suggested that the antimycobacterial activity may be mediated through inhibition of the RNA polymerase-binding protein RbpA, a transcriptional regulator essential for mycobacterial survival, with two propanoate compounds showing binding energies of −5.4 and −6.3 kcal/mol [81]. Plant-derived compounds that can prevent biofilm formation on medical devices or disperse established biofilms represent a valuable adjunct to conventional antibiotic therapy. However, the translation of these findings to clinical practice requires standardized methods for evaluating anti-biofilm activity and a better understanding of the pharmacokinetic properties required to achieve effective concentrations at biofilm sites in vivo [59,61]. Collectively, these findings indicate that plant-derived antibiofilm agents offer a multi-pronged attack on biofilm-associated infections, yet the field would benefit from standardized protocols for evaluating antibiofilm activity and from studies conducted in physiologically relevant models.

### 2.3. Interference with Quorum Sensing (QS) Systems

Quorum sensing (QS) is a cell-to-cell communication system employed by bacteria to coordinate population-level behaviors, including virulence factor expression, biofilm maturation, and antibiotic tolerance, in response to fluctuations in cell density [82,83]. This signaling is mediated by the production, secretion, and detection of small diffusible molecules known as autoinducers, such as N-acyl homoserine lactones (AHLs) in Gram-negative bacteria and autoinducing peptides (AIPs) in Gram-positive bacteria [84,85]. The central role of QS in regulating virulence without affecting bacterial growth has positioned QS inhibition as a particularly attractive antivirulence strategy that may exert reduced selective pressure for the development of resistance compared to conventional bactericidal agents [86,87].

Plants have evolved sophisticated chemical defense mechanisms to combat bacterial pathogens, and many of these involve the production of QS-interfering compounds that disrupt bacterial communication [88,89]. Ajoene, a sulfur-containing compound derived from garlic (*Allium sativum*), represents one of the most extensively characterized plant-derived QS inhibitors. Jakobsen et al. (2012) demonstrated that ajoene specifically inhibits genes controlled by QS in *P. aeruginosa*, reducing the production of virulence factors such as rhamnolipids and elastase and rendering biofilms more susceptible to antibiotic treatment and immune clearance. Notably, transcriptomic analysis revealed that ajoene downregulates only a small subset of the QS regulon (11 genes at 80 μg/mL), primarily targeting the Rhl system rather than acting as a broad-spectrum QS antagonist. The expression of rhlA, encoding a rhamnosyltransferase essential for rhamnolipid biosynthesis, was reduced almost 12-fold, resulting in near-complete abrogation of rhamnolipid production at 80 μg/mL [90]. This targeted mechanism contrasts with that of other QS inhibitors such as furanone C-30, which downregulates more than 80% of the QS-controlled genes, suggesting that ajoene may act at the posttranscriptional level, possibly through interference with the Hfq protein or small regulatory RNAs [90].

The mechanism of action involves the inhibition of the las and rhl QS systems through interaction with the regulatory proteins LasR and RhlR [85,91]. Beyond ajoene, other hydrophobic garlic compounds contribute to QS inhibition: allicin, the most abundant sulfur-containing compound in raw garlic, prevents biofilm formation by inhibiting early bacterial adhesion and EPS secretion, while diallyl disulfide (DAS2) suppresses the expression of key QS-related genes in *Pseudomonas* spp. at subinhibitory concentrations (0.16–1.28 mg/mL) without affecting microbial growth [91]. However, the clinical utility of these compounds is constrained by their chemical instability, particularly in blood, limiting their current therapeutic potential to topical or inhalatory applications [91].

Flavonoids have also demonstrated appreciable QS inhibitory activity. Packiavathy et al. (2014) showed that curcumin, the principal curcuminoid from *Curcuma longa*, inhibited biofilm development in uropathogens through an anti-QS mechanism, reducing the production of AHLs and downregulating QS-dependent genes [92]. This finding is consistent with the broader anti-infective potential of curcumin against multiple pathogens, including *Mycobacterium tuberculosis* [93,94]. Beyond curcumin, a comprehensive review of South African medicinal plants has identified numerous antimycobacterial compounds with activity against drug-resistant strains, highlighting the untapped potential of ethnopharmacological knowledge in the search for novel anti-infective agents [95]. Among the 117 compounds catalogued, the naphthoquinones 7-methyljuglone and juglone, isolated from *Euclea natalensis*, exhibited potent activity against *M. tuberculosis* with MIC values of 0.5 and 1.0 μg/mL, respectively. Notably, 7-methyljuglone demonstrated synergy with both isoniazid (FIC index 0.5) and rifampicin (FIC index 0.2), reducing the MIC of these first-line antitubercular drugs by 4–6 fold. The proposed mechanism involves subversive substrate activity against mycothiol disulfide reductase (Mtr), a validated and mycobacterium-specific drug target with no human orthologue, highlighting the potential of plant-derived compounds to engage novel targets and potentiate existing antibiotics [95].

Prabhakaran et al. (2025) reported that plant-based O-methylated flavonoids effectively disrupted QS and biofilm formation in *P. aeruginosa*, with molecular docking studies suggesting that these compounds act as competitive antagonists of QS receptors [61]. Qaralleh (2023) evaluated the QS inhibitory effect of *Nepeta curviflora* methanolic extract against extended-spectrum β-lactamase (ESBL)-producing *P. aeruginosa*, demonstrating significant attenuation of QS-regulated phenotypes including swarming motility, pyocyanin production, and biofilm formation. At sub-inhibitory concentrations (1/4 MIC, 2.5 mg/mL), the extract reduced pyocyanin production by 76.5%, rhamnolipid production by 64.3%, and biofilm formation by 66.3%, while also significantly decreasing AHL secretion (66.6% inhibition), swarming motility, and exopolysaccharide production. LC-MS analysis identified kaempferol (17.4%), quercetin (15.3%), salicylic acid (10.5%), rutin (8.1%), and rosmarinic acid (7.3%) as the major constituents, suggesting that the anti-QS activity is attributable to the synergistic action of flavonoid and phenolic compounds [96].

Beyond their in vitro activity, certain QS inhibitors have demonstrated promising effects in in vivo models. For instance, vanillin (the primary component of vanilla bean extract from *Vanilla planifolia*) and umbelliferone (a coumarin derivative found in plants of the Apiaceae family such as coriander and fennel) were shown by Deryabin et al. (2023) to modulate the cecal microbiome and reduce inflammation in a broiler chicken model, providing a concrete example of how phytochemical QS inhibitors can exert beneficial effects in a live animal system relevant to the One Health framework [97]. The antivirulence paradigm underlying QS inhibition offers several theoretical advantages over conventional bactericidal approaches. By targeting non-essential regulatory systems rather than growth-essential processes, QS inhibitors may impose weaker selective pressure for resistance development [32,86]. This concept was initially articulated in the context of aquaculture, where disruption of bacterial cell-to-cell communication was proposed as an alternative to conventional antibiotics, with the rationale that such strategies pose selective pressure only under those environmental conditions where virulence is essential, in contrast to antibiotics, which exert strong selective pressure in any environment [98]. This ecological perspective is equally relevant to human pathogens, where the conditional nature of virulence regulation may similarly limit the emergence and spread of resistance to QS-disrupting therapies [86,98]. Defoirdt (2025) provided evidence that resistance to QS inhibition spreads more slowly during host infection than antibiotic resistance, with no spread observed when resistance-conferring mutations are initially rare, supporting the potential of these strategies for sustainable antimicrobial therapy. Furthermore, QS inhibitors can be used at sub-inhibitory concentrations that do not affect bacterial growth, potentially reducing off-target effects on the host microbiome [97,99]. The potential of phytochemicals to reduce antibiotic dependence extends beyond human medicine; in poultry production, plant-derived compounds have shown promise as antibiotic alternatives against bacterial foodborne pathogens, contributing to the One Health framework for combating antimicrobial resistance [100].

The plant kingdom offers an enormous reservoir of QS-inhibitory compounds with diverse chemical scaffolds. Pérez-Montaño et al. (2013) demonstrated that rice and bean plants produce AHL-mimic QS signals that specifically interfere with biofilm formation by plant-associated bacteria, suggesting that QS inhibition is a natural component of plant defense against bacterial pathogens [101]. This evolutionary arms race between plants and bacteria has generated a rich diversity of QS-modulatory molecules that can be exploited for therapeutic purposes [89,102]. The identification and characterization of these compounds, coupled with medicinal chemistry efforts to optimize their potency and selectivity, represent a promising avenue for the development of next-generation anti-infective therapies that disarm rather than kill bacterial pathogens [84,103]. From a mechanistic perspective, plant-derived QS inhibitors can be broadly classified into three categories based on their mode of action: (i) specific inhibitors that directly bind to LuxI-type AHL synthases and/or LuxR-type receptor proteins, as demonstrated for terpenes (carvacrol), phenylpropanoids (cinnamaldehyde, eugenol), and flavonoids (quercetin); (ii) non-specific inhibitors that affect QS-related intracellular regulatory pathways, such as sulfur-containing compounds (ajoene, iberin) that lower the expression of regulatory small RNAs; and (iii) indirect inhibitors that alter metabolic pathways involved in QS-dependent processes, as observed for vanillic acid and curcumin [85]. Overall, this mechanistic classification underscores the versatility of plant-derived QS inhibitors and suggests that combining compounds with different modes of action may achieve more robust and durable QS blockade than single-agent strategies.

Table 4 provides a detailed overview of plant-derived quorum sensing inhibitors, their specific molecular targets within QS systems, and their antivirulence effects on ESKAPE pathogens.

### 2.4. Membrane Permeabilization and Cell Wall Damage

The bacterial cell envelope constitutes the primary barrier that antibiotics must overcome to reach their intracellular targets. In Gram-negative ESKAPE pathogens, the outer membrane represents an additional permeability barrier that restricts the entry of many antibiotics, contributing to intrinsic and acquired resistance [12,104]. Plant-derived compounds that disrupt membrane integrity or increase membrane permeability can therefore function as antibiotic adjuvants by facilitating the entry of otherwise ineffective antibiotics into the bacterial cell [53,105].

Essential oils and their monoterpene constituents are among the most effective membrane-active plant compounds. The lipophilic nature of these molecules enables them to partition into the bacterial membrane, where they increase fluidity, disrupt lipid packing, and cause leakage of ions and cellular contents [74,75]. The resulting membrane damage can directly cause cell death at high concentrations, while at sub-inhibitory concentrations, it can synergize with conventional antibiotics by increasing their intracellular accumulation [106,107]. Gorlenko et al. (2020) reviewed plant secondary metabolites active against drug-resistant bacteria and emphasized the importance of membrane-active compounds as adjuvants that can potentiate multiple classes of antibiotics simultaneously, reducing the likelihood of resistance development [37].

Among the most extensively studied membrane-active monoterpenes, terpinen-4-ol, the major component of tea tree oil (*Melaleuca alternifolia*), has been shown to disrupt membrane integrity in *S. aureus* and *P. aeruginosa*, leading to leakage of intracellular ions and metabolites, and potentiating the activity of aminoglycosides and beta-lactams [108]. Nerol, an acyclic monoterpene found in lemongrass (*Cymbopogon citratus*) and other essential oils, exhibits similar membrane-disrupting properties, with studies demonstrating synergistic effects with fluoroquinolones against MDR Gram-negative bacteria [109]. Beyond terpenes, the phenylpropene eugenol, a major constituent of clove oil (*Syzygium aromaticum*), has been demonstrated to permeabilize the outer membrane of Gram-negative bacteria, enhancing the intracellular accumulation of co-administered antibiotics such as ampicillin and erythromycin, to which these bacteria are normally resistant [110,111]. Furthermore, baicalin, a flavonoid glycoside from *Scutellaria baicalensis* (Chinese skullcap), exemplifies a multi-mechanistic adjuvant, having been reported to potentiate antibiotics not only by disrupting membrane function but also by inhibiting biofilm formation and efflux pumps in MDR pathogens [106,112]. These diverse membrane-active phytochemicals offer a rich source of potential adjuvants capable of restoring antibiotic susceptibility across multiple ESKAPE pathogens.

The antimicrobial peptides and proteins found in plant extracts represent another important class of membrane-active compounds [113,114]. Although less extensively studied than essential oils, these molecules often exhibit selectivity for bacterial membranes over mammalian cell membranes due to differences in lipid composition and membrane potential [115,116]. Importantly, the mechanism of membrane perturbation by antimicrobial peptides can vary considerably depending on the target bacterial strain, as demonstrated by Wang et al. (2023), who showed that the same peptide can act as a potent pore-former in one species while exhibiting only subtle membrane depolarization in another. This strain-dependent variability underscores the importance of employing multiple complementary assays, including measurements of membrane potential, intracellular pH, and ATP leakage, when characterizing the membrane-active properties of plant-derived antimicrobial compounds [113]. de Jesus Dzul-Beh et al. (2023) investigated the antimicrobial potential of *Matayba oppositifolia*, a Mayan medicinal plant traditionally used for diarrhea and skin sores, against drug-susceptible and drug-resistant ESKAPE-E pathogens. The n-hexane bark extract exhibited the most potent activity, particularly against carbapenem-resistant *K. pneumoniae* (MIC = 31.25–500 μg/mL) and *A. baumannii* (MIC = 125–250 μg/mL), including XDR strains. GC-MS analysis identified 12 bioactive compounds, with palmitic acid, friedelan-3-one, and 7-dehydrodiosgenin as the major constituents, suggesting that the antimicrobial activity may be attributed to membrane-disrupting fatty acids, terpenes, and steroids [117].

The membrane-active phytochemicals discussed in this section, along with their plant sources, target ESKAPE pathogens, and mechanisms of action, are summarized in Table 5.

The combination of membrane-active phytochemicals with antibiotics that are normally excluded by the outer membrane of Gram-negative bacteria represents a particularly promising strategy. By transiently permeabilizing the outer membrane, plant-derived compounds can restore the susceptibility of MDR pathogens to antibiotics that would otherwise be ineffective [36,54]. This approach has been demonstrated for combinations of essential oil components with hydrophobic antibiotics such as macrolides and rifampicin against *P. aeruginosa* and *A. baumannii* [48,75]. Importantly, the membrane-permeabilizing effects of many phytochemicals are concentration-dependent and reversible, allowing for controlled antibiotic entry without permanent damage to host cell membranes [25,53].

Recent advances in nanotechnology have opened new possibilities for enhancing the membrane-targeting properties of plant-derived compounds. Nanoformulations of essential oils and other hydrophobic phytochemicals can improve their aqueous solubility, stability, and selective delivery to bacterial membranes, potentially increasing their therapeutic index and clinical applicability [118,119]. The development of such advanced delivery systems, discussed in detail in Section 4.4, represents a critical step toward translating the membrane-active properties of phytochemicals from in vitro observations to in vivo therapeutic applications.

## 3. Synergistic Strategies: Combining Phytochemicals with Conventional Antibiotics

The identification of plant-derived compounds that inhibit resistance mechanisms or attenuate virulence represents only the first step toward their therapeutic application. The translation of these findings into clinically relevant strategies requires the systematic evaluation of how phytochemical adjuvants interact with conventional antibiotics when used in combination. Synergy, defined as a combined effect that is significantly greater than the sum of the individual effects of each agent alone, is the ideal outcome of such combinations, as it allows for dose reduction of both the antibiotic and the adjuvant while achieving equivalent or superior antibacterial efficacy [36,120].

The pursuit of synergistic combinations between phytochemicals and conventional antibiotics represents a promising strategy to restore the activity of existing antibiotics against MDR ESKAPE pathogens while potentially reducing toxicity and slowing the emergence of further resistance [19,22].

### 3.1. Methodologies for Evaluating Synergy

The rigorous and standardized evaluation of synergistic interactions is essential for the credibility and reproducibility of research on antibiotic adjuvants. Several established methodologies are employed to quantify the nature and magnitude of interactions between phytochemicals and conventional antibiotics, each with distinct advantages and limitations [121,122].

The checkerboard assay represents the most widely used method for initial synergy screening. This technique involves the preparation of a two-dimensional matrix of concentrations of two antimicrobial agents, typically in a 96-well microtiter plate format, allowing for the simultaneous testing of multiple concentration combinations [120,122]. From the checkerboard data, the Fractional Inhibitory Concentration Index (FICI) is calculated as the sum of the FICs of each agent, where the FIC of an individual agent is defined as its MIC in combination divided by its MIC alone. A FICI ≤ 0.5 is generally interpreted as synergistic, a FICI between 0.5 and 4.0 as indifferent or additive, and a FICI > 4.0 as antagonistic [36,120]. For adjuvants that lack intrinsic antimicrobial activity, Wright (2016) proposed an additional parameter, the Rescue Concentration (RC), defined as the concentration of adjuvant required to reduce the antibiotic MIC in resistant bacteria to a value equal to or below the clinical breakpoint. This metric offers a clinically relevant benchmark for prioritizing adjuvant candidates, as it directly addresses the question of whether the combination can restore antibiotic susceptibility at therapeutically achievable concentrations [18]. Despite its widespread use, the checkerboard assay has limitations, including its inability to distinguish between bacteriostatic and bactericidal interactions and its reliance on a single time point of evaluation [121]. Furthermore, the interpretation of membrane-active compounds requires particular care, as the observed effects can vary substantially depending on the bacterial strain and the specific assay employed [113]. For instance, Wang et al. (2023) demonstrated that the lantibiotic nisin, a well-characterized pore-former, showed expected severe membrane disruption in *Staphylococcus simulans* and *Bacillus megaterium*, yet produced anomalous results in *Micrococcus flavus*, where the pH gradient was only minimally affected despite rapid killing. Such findings highlight the necessity of using multiple bacterial strains and complementary assays when evaluating membrane-active phytochemical adjuvants, to avoid misinterpretation of their mechanisms of action [113].

Time-kill kinetics provides a more dynamic and informative assessment of synergistic interactions by monitoring the rate and extent of bacterial killing over time in the presence of individual agents and their combinations [122,123]. Synergy in time-kill assays is typically defined as a ≥2 log10 CFU/mL decrease in viable bacterial counts for the combination compared to the most active single agent at a specified time point, usually 24 h. This method offers the advantage of capturing the temporal dynamics of antibacterial activity and distinguishing between concentration-dependent and time-dependent killing, parameters that are critical for optimizing dosing regimens [22,121].

Additional methods for evaluating synergy include the E-test overlay method, which can provide a rapid qualitative assessment of interactions, and the use of isobolograms, which graphically represent the concentrations of two agents that produce a defined effect [36,120]. More recently, advanced methodologies such as high-throughput screening platforms and computational modeling have been applied to accelerate the identification of synergistic phytochemical-antibiotic combinations [49,107]. The adoption of standardized methodologies across studies is essential to ensure the comparability of results and to facilitate the translation of promising combinations from in vitro observation to in vivo validation [22,124].

### 3.2. Re-Sensitizing ESKAPE Pathogens to First-Line Therapies

The ultimate goal of combining phytochemical adjuvants with conventional antibiotics is to re-sensitize MDR ESKAPE pathogens to first-line therapies that have become ineffective due to the acquisition of resistance mechanisms [18,29]. Numerous studies have documented the ability of plant-derived compounds to restore the activity of critically important antibiotics against ESKAPE pathogens, often reducing the MIC of the antibiotic by several orders of magnitude [31,49].

Table 6 summarizes representative synergistic combinations between plant-derived compounds and conventional antibiotics against ESKAPE pathogens, highlighting the magnitude of synergy and the underlying mechanisms where known.

Flavonoids have demonstrated consistent synergy with fluoroquinolones against *S. aureus*, including MRSA strains. The co-administration of quercetin or kaempferol with ciprofloxacin or norfloxacin results in significant reductions in the MIC of the fluoroquinolone through inhibition of the NorA efflux pump, which is the primary mechanism of fluoroquinolone resistance in this pathogen [15,49]. The FICI values for these combinations are frequently in the synergistic range (≤0.5), and time-kill studies have confirmed that the combination is bactericidal at concentrations at which the individual agents are merely bacteriostatic or inactive [15,22].

Against Gram-negative ESKAPE pathogens, the combination of essential oil components with antibiotics that are normally excluded by the outer membrane has yielded particularly promising results. Carvacrol and thymol, in combination with antibiotics such as colistin, rifampicin, or macrolides, have demonstrated strong synergy against *A. baumannii*, *P. aeruginosa*, and *K. pneumoniae* [48,75]. The mechanism underlying this synergy involves the permeabilization of the outer membrane by the lipophilic monoterpenes, which facilitates the entry of the co-administered antibiotic into the bacterial cell [53,106]. Beyond membrane permeabilization, traditional Chinese medicine (TCM) monomers have been shown to act through multiple adjuvant mechanisms. For instance, baicalein potentiates tetracycline against MRSA by inhibiting the Tet(K) efflux pump (FICI 0.03125–0.125), while berberine hydrochloride combined with tigecycline, sulbactam, meropenem, or ciprofloxacin reduces the efflux of antibiotics in *A. baumannii* through downregulation of the adeB efflux pump gene (FICI < 0.5) [106]. Furthermore, flavonoids such as myricetin, 7,8-dihydroxyflavone, and luteolin disrupt bacterial iron homeostasis to potentiate colistin efficacy against Gram-negative pathogens (FICI 0.125–0.458), a novel mechanism that enhances colistin binding and promotes lethal ROS production [106]. Importantly, the membrane-permeabilizing effects of essential oil components are often reversible and concentration-dependent, allowing for a transient increase in antibiotic uptake without causing irreversible damage to host cell membranes [25,75].

The re-sensitization of biofilm-embedded bacteria represents an additional clinical scenario in which phytochemical–antibiotic combinations may offer notable advantages. As discussed in Section 2.2, biofilm formation confers tolerance to antibiotics at concentrations far exceeding those achievable in vivo. Plant-derived compounds that disrupt biofilm structure or inhibit QS-regulated biofilm maintenance can restore antibiotic susceptibility in previously tolerant cells [60,61]. For example, ajoene in combination with tobramycin has been shown to eradicate *P. aeruginosa* biofilms that were refractory to tobramycin alone, an effect attributed to the QS-inhibitory activity of ajoene that renders biofilm cells metabolically active and thus susceptible to the bactericidal action of the aminoglycoside [90,102].

The clinical potential of these synergistic combinations is further supported by the concept of “phytochemical synergy,” in which multiple compounds within a plant extract act on different targets simultaneously, producing an effect greater than that of any single isolated compound [35,125]. This multi-target approach mirrors the complexity of bacterial resistance mechanisms and may reduce the likelihood of resistance emergence, as bacteria would need to simultaneously overcome multiple inhibitory mechanisms [32,86]. However, the complexity of plant extracts also poses challenges for standardization and quality control, issues that must be addressed to advance these combinations toward clinical application [29,34].

Despite the abundance of in vitro evidence supporting the synergistic potential of phytochemical–antibiotic combinations, the number of studies that have progressed to in vivo validation remains limited [126,127]. The translational gap between in vitro synergy and in vivo efficacy represents the most significant obstacle to the clinical development of these combinations and is the focus of Section 4. The establishment of robust in vivo models, the optimization of pharmacokinetic compatibility between adjuvants and antibiotics, and the development of appropriate formulations that ensure adequate bioavailability at the site of infection are critical priorities for future research [128,129]. In summary, while the in vitro synergy data are compelling, the scarcity of in vivo confirmation remains the Achilles’ heel of this field and should be prioritized in future investigations.

## 4. Translational Challenges and Future Perspectives

**Figure 2:**
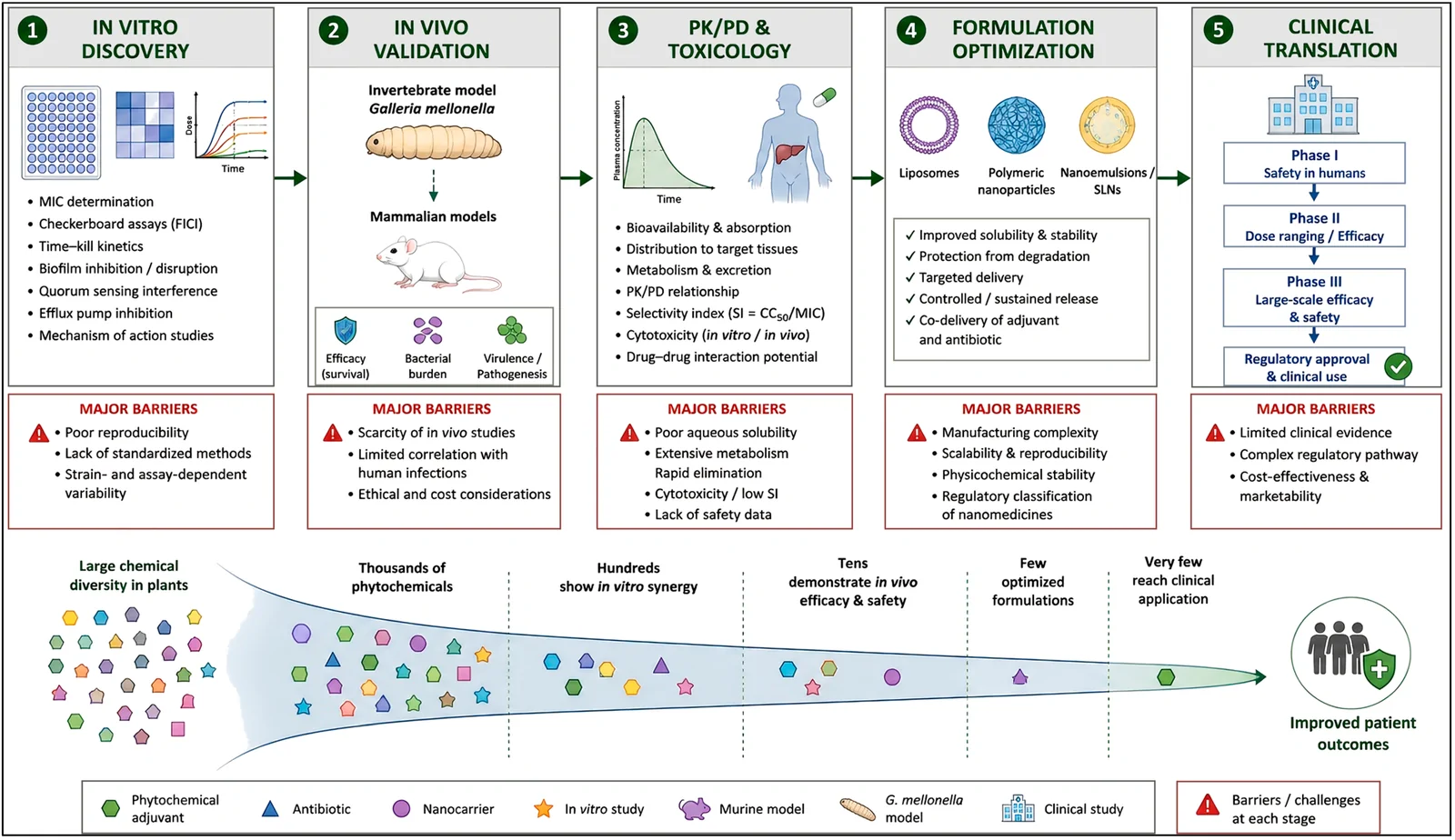
Translational pipeline for the development of plant-derived antibiotic adjuvants against multidrug-resistant ESKAPE pathogens. The development of phytochemical adjuvants follows a stepwise translational pathway encompassing (**1**) in vitro identification of synergistic activity and mechanism of action, (**2**) validation in invertebrate and mammalian infection models, (**3**) pharmacokinetic, pharmacodynamic, and toxicological characterization, (**4**) formulation optimization through advanced delivery systems such as liposomes, polymeric nanoparticles, and nanoemulsions, and (**5**) clinical evaluation and regulatory approval. Major barriers associated with each stage—including poor bioavailability, limited in vivo validation, cytotoxicity concerns, lack of standardized extracts, formulation challenges, and regulatory constraints—are highlighted in red, illustrating the principal obstacles that currently limit the clinical translation of plant-derived antibiotic adjuvants. Abbreviations: PK/PD: Pharmacokinetics/Pharmacodynamics; SI: Selectivity Index; CC_50_: 50% cytotoxic concentration; MIC: Minimum inhibitory concentration; SLNs: Solid lipid nanoparticles.

The substantial body of in vitro evidence demonstrating the capacity of plant-derived compounds to function as antibiotic adjuvants against MDR ESKAPE pathogens has not yet translated into clinically approved therapies [18,29]. The major stages and barriers along this translational pipeline are summarized in Figure 2. This “translational gap” reflects a series of interconnected challenges that must be systematically addressed to advance phytochemical adjuvants from the laboratory to the bedside. Notably, even conventional antibiotics in development face formidable resistance challenges: Daruka et al. (2025) demonstrated that 13 antibiotics introduced after 2017 or currently in clinical development showed similar susceptibility to resistance evolution as antibiotics that have been in use for decades, with resistance-conferring mutations already prevalent in natural pathogen populations. This finding reinforces the value of antibiotic adjuvants, including plant-derived compounds, as orthogonal strategies that may help preserve the efficacy of both existing and future antibiotics by targeting resistance mechanisms rather than essential bacterial processes [9]. These challenges include issues related to pharmacokinetics and bioavailability, standardization of plant extracts, potential toxicity and selectivity concerns, and the scarcity of robust in vivo models that recapitulate the complexity of human infections [30,33,54]. This section critically examines these barriers and discusses emerging strategies, including nanotechnology-based delivery systems and advanced in vivo models, that may facilitate the clinical translation of plant-derived antibiotic adjuvants.

The principal translational challenges facing the development of plant-derived antibiotic adjuvants, along with their descriptions and potential mitigation strategies, are summarized in Table 7.

### 4.1. Pharmacokinetics, Bioavailability, and Standardization Issues

One of the most significant obstacles to the clinical development of phytochemical adjuvants is their unfavorable pharmacokinetic profile. Many plant-derived compounds with potent in vitro activity, including flavonoids, terpenes, and polyphenols, exhibit poor aqueous solubility, limited gastrointestinal absorption, extensive first-pass metabolism, and rapid systemic elimination [33,34].

These pharmacokinetic limitations result in plasma and tissue concentrations that are often orders of magnitude lower than the in vitro effective concentrations required for efflux pump inhibition, quorum sensing interference, or membrane permeabilization [15,48]. For example, the flavonoid quercetin, despite its well-documented in vitro EPI activity against NorA in *S. aureus*, undergoes extensive glucuronidation and sulfation in the intestinal epithelium and liver, resulting in very low systemic bioavailability of the parent compound [15,33]. Similarly, curcumin, a polyphenol with well-characterized antimycobacterial, antibiofilm, and quorum sensing-inhibitory activities, suffers from extremely poor aqueous solubility and rapid systemic metabolism, prompting the development of advanced delivery systems such as nanoparticles, liposomes, and phospholipid complexes to enhance its bioavailability and therapeutic efficacy [93].

The bioavailability challenge is compounded by the fact that many phytochemical adjuvants are intended to act at the site of infection, which may be located in tissues with limited drug penetration, such as biofilms on medical devices, the lungs in cystic fibrosis patients, or intracellular compartments where certain ESKAPE pathogens can reside [56,126]. Achieving and maintaining effective adjuvant concentrations at these sites requires not only adequate systemic absorption but also appropriate tissue distribution and sufficient residence time at the target site [34,48].

A systematic consideration of the Absorption, Distribution, Metabolism, and Excretion (ADME) properties of phytochemical adjuvants is essential to understand their pharmacokinetic limitations and to guide formulation strategies. Although comprehensive ADME data are available for only a limited subset of phytochemicals, the existing evidence reveals common patterns that inform translational development.

Absorption: The oral bioavailability of most flavonoid and polyphenolic phytochemicals is severely limited by poor aqueous solubility and extensive first-pass metabolism. Quercetin, for instance, exhibits an oral bioavailability of less than 2% in humans due to its low solubility and rapid conjugation in the intestinal epithelium, although formulation as phytosomes or nanoparticles has been shown to increase absorption by up to 20-fold [33,136]. Similarly, curcumin demonstrates negligible systemic absorption following oral administration, with peak plasma concentrations rarely exceeding 0.5 μM even at doses of several grams, a limitation that has driven the development of liposomal and phospholipid-complexed formulations [93,137]. Essential oil components such as carvacrol and thymol show more favorable absorption profiles due to their lipophilic nature, although they undergo rapid hepatic metabolism [50,74].

Distribution: The tissue distribution of phytochemical adjuvants is a critical determinant of their ability to reach the site of infection. Many flavonoids and polyphenols exhibit extensive plasma protein binding, which limits their free fraction available for tissue penetration [33,138]. Curcumin, for example, shows preferential accumulation in the liver and intestinal mucosa following oral administration, with limited distribution to extrahepatic tissues, although nanoparticle formulations have been shown to enhance its tissue penetration, including across the blood–brain barrier [93,137]. Essential oil components, due to their high lipophilicity, distribute rapidly into tissues and can achieve therapeutic concentrations in the lungs, skin, and urinary tract, making them particularly suitable for topical and inhalatory applications [74,108]. However, achieving effective concentrations within biofilms, where diffusion is severely restricted by the EPS matrix, remains a significant challenge for all phytochemical classes [56].

Metabolism: Phase II conjugation reactions, particularly glucuronidation and sulfation, represent the predominant metabolic pathways for flavonoids and polyphenols, resulting in the rapid conversion of parent compounds to inactive or significantly less active metabolites [33,136]. Quercetin is extensively metabolized to quercetin-3-glucuronide and quercetin-3′-sulfate, which retain only minimal efflux pump inhibitory activity compared to the parent aglycone [33]. Similarly, curcumin undergoes rapid reduction and conjugation to tetrahydrocurcumin and curcumin glucuronides, which have substantially reduced antibacterial and antibiofilm activities [93]. Terpenes and essential oil components are primarily metabolized by cytochrome P450 enzymes and undergo glucuronidation prior to urinary excretion [50,74]. The use of bioenhancers such as piperine, which inhibits glucuronidation, has been shown to significantly increase the bioavailability of co-administered phytochemicals, including curcumin, by up to 2000% [137].

Excretion: The majority of phytochemical adjuvants and their metabolites are excreted via the biliary and renal routes, with elimination half-lives typically ranging from 1 to 4 h for most flavonoids and terpenes [33,136]. This rapid elimination necessitates frequent dosing or the use of sustained-release formulations to maintain therapeutic concentrations at the site of infection. Notably, certain phytochemicals, including curcumin and resveratrol, undergo significant enterohepatic recirculation, which can prolong their residence time in the body but also complicates pharmacokinetic modeling [93,137]. The development of nanoformulations with prolonged circulation times, such as PEGylated liposomes and polymeric nanoparticles, represents a promising strategy to extend the elimination half-life of rapidly cleared phytochemicals and to reduce dosing frequency [118,119].

The systematic evaluation of ADME properties for each candidate phytochemical adjuvant is essential to prioritize compounds with favorable pharmacokinetic profiles for further development and to design appropriate formulation strategies to overcome the inherent limitations of this compound class.

A further complicating factor is the inherent complexity and variability of plant extracts. Unlike single-molecule antibiotics, plant extracts are complex mixtures of numerous bioactive compounds whose composition can vary significantly depending on the plant species, geographic origin, cultivation conditions, harvest time, and extraction method [35,130]. This variability poses substantial challenges for standardization, quality control, and batch-to-batch reproducibility, all of which are essential requirements for regulatory approval and clinical use [25,29]. While the isolation and purification of single active compounds can address some of these standardization issues, this approach may sacrifice the “phytochemical synergy” that often underlies the superior activity of whole extracts compared to isolated constituents [35,125]. As noted by Gibbons (2008), this intrinsic polypharmacology represents a potentially valuable therapeutic innovation; however, this same complexity poses unprecedented phytochemical challenges, demanding rigorous chromatographic or spectroscopic profiling to ensure batch-to-batch conformity, correct taxonomic identification of source material, and comprehensive toxicological evaluation to guarantee safety [29].

Beyond pharmacokinetic and standardization challenges, the development of plant-derived antibiotic adjuvants faces significant economic hurdles that are often underappreciated in the academic literature. The pharmaceutical industry has historically shown limited interest in natural product research due to several factors: the difficulty of obtaining intellectual property protection for naturally occurring compounds, the high costs associated with complex purification and standardization processes, and the relatively small market size for agents that are designed to be used exclusively in combination with existing, often generic, antibiotics [132]. Unlike novel antibiotics, which can command high prices justified by their life-saving potential in last-resort scenarios, antibiotic adjuvants face uncertain reimbursement pathways. Moreover, the return on investment for natural product-based adjuvant therapies is further constrained by the fact that many of the most promising phytochemicals, such as quercetin, curcumin, and various essential oil components, are widely available as low-cost dietary supplements, potentially discouraging the substantial financial investment required for clinical trials and regulatory approval [132,133]. These economic realities, when combined with the scientific challenges discussed above, create a formidable barrier to the translation of phytochemical adjuvants from bench to bedside, and underscore the need for alternative funding models, such as public–private partnerships and non-profit drug development initiatives, to advance this promising but commercially challenging field.

This concept is well illustrated by berberine, a benzylisoquinoline alkaloid from *Coptis chinensis* and other medicinal plants, which has been shown to act simultaneously as an efflux pump inhibitor (downregulating MexXY-OprM in *P. aeruginosa* and AdeB in *A. baumannii*), a biofilm disruptor, and a membrane permeabilizer, potentiating multiple classes of antibiotics including aminoglycosides, β-lactams, and fluoroquinolones [106]. Such multi-target adjuvants exemplify the therapeutic potential of phytochemical synergy, but also underscore the challenges of isolating and standardizing the individual active components responsible for these pleiotropic effects.

Strategies to overcome these pharmacokinetic and standardization challenges include the development of standardized extracts with defined chemical fingerprints, the use of bioenhancers that improve the absorption of co-administered phytochemicals, and the synthesis of more stable and bioavailable structural analogs of active natural compounds [29,36]. Furthermore, the application of nanotechnology and advanced drug delivery systems, discussed in detail in Section 4.4, offers promising avenues to improve the solubility, stability, and targeted delivery of phytochemical adjuvants [114,118].

### 4.2. Stability Considerations for Phytochemical Formulations

In addition to pharmacokinetic limitations, the stability of plant-derived formulations represents a critical yet often overlooked factor influencing their therapeutic potential and clinical translatability. Phytochemicals are susceptible to degradation through multiple pathways, including oxidation, hydrolysis, photodegradation, and thermal decomposition, which can significantly reduce their bioactivity during storage, processing, and administration [139]. The stability profile of individual phytochemicals varies considerably depending on their chemical structure and the environmental conditions to which they are exposed.

Flavonoids such as quercetin and EGCG are particularly susceptible to oxidative degradation, especially in aqueous solutions at neutral to alkaline pH, where they undergo rapid autoxidation leading to the formation of quinones and other degradation products with reduced or altered biological activity [139,140]. Quercetin, for instance, shows significant degradation within 24 h in phosphate buffer at pH 7.4, with accelerated decomposition upon exposure to light and elevated temperatures [140]. Curcumin is notoriously unstable under physiological conditions, undergoing rapid hydrolytic degradation at pH above 7.0, with a half-life of less than 30 min in phosphate-buffered saline, which severely limits its therapeutic application [137,141]. Essential oil components such as carvacrol, thymol, and terpinen-4-ol are volatile and susceptible to oxidation and photodegradation, requiring protection from light and oxygen during storage [74,142]. Furthermore, many phytochemicals can interact with excipients commonly used in pharmaceutical formulations, leading to incompatibility issues that affect both stability and bioavailability [139].

Several strategies have been developed to enhance the stability of phytochemical formulations, many of which overlap with approaches to improve bioavailability. Encapsulation within nanocarriers such as liposomes, solid lipid nanoparticles (SLNs), and polymeric nanoparticles provides a physical barrier that protects labile phytochemicals from environmental degradation [118,139]. For example, curcumin encapsulated in PLGA nanoparticles exhibits significantly enhanced stability at physiological pH, with a half-life extended from minutes to several hours [141]. Similarly, liposomal encapsulation of quercetin protects the flavonoid from oxidative degradation and maintains its efflux pump inhibitory activity during prolonged storage [140]. Lyophilization (freeze-drying) of phytochemical formulations can further enhance long-term stability by removing water and preventing hydrolytic degradation, although the process parameters must be carefully optimized to avoid damaging the active compounds [139,142].

The use of antioxidants such as ascorbic acid, butylated hydroxytoluene (BHT), and α-tocopherol as stabilizing excipients has also been shown to reduce oxidative degradation of sensitive phytochemicals during storage [139]. For essential oils and other volatile compounds, storage under inert atmospheres (e.g., nitrogen or argon), protection from light using amber or opaque containers, and maintenance of low temperatures (4 °C or below) are recommended to minimize degradation [74,142]. For clinical translation, the development of stable, ready-to-use formulations with defined shelf-lives and storage conditions is essential, and stability testing according to International Council for Harmonisation (ICH) guidelines should be incorporated early in the development process [139].

### 4.3. Toxicity, Cytotoxicity, and the Selectivity Index

A critical consideration in the development of any new therapeutic agent is the balance between efficacy and toxicity, often quantified as the Selectivity Index (SI), which is the ratio of the concentration at which the compound is toxic to mammalian cells (CC50 or IC50) to the concentration at which it is effective against the target pathogen (MIC or EC50) [33,54]. For antibiotic adjuvants, a high SI is particularly important, as these compounds are intended to be co-administered with conventional antibiotics, and any adjuvant-induced toxicity could limit the overall therapeutic window of the combination [18,44].

Many plant-derived compounds have demonstrated potent in vitro antibacterial or resistance-modifying activity, but their toxicity profile has not been adequately characterized. Cushnie and Lamb (2005) reviewed the antimicrobial activity of flavonoids and noted that while many flavonoids exhibit low acute toxicity, their chronic toxicity, potential for drug interactions, and effects on human cytochrome P450 enzymes require further investigation. Similarly, essential oil components such as carvacrol and thymol, despite their impressive membrane-permeabilizing and antibiofilm activities, can be cytotoxic to mammalian cells at concentrations only moderately higher than their effective antibacterial concentrations, potentially limiting their therapeutic index [74,75]. An illustrative example of this delicate balance is provided by berberine, a broad-spectrum plant alkaloid whose potent intrinsic antibacterial activity is unfortunately accompanied by significant toxicity, a problem that adjuvant-based strategies seek to overcome by employing such compounds at sub-therapeutic concentrations to modulate resistance rather than to directly kill bacteria [29]. Another telling example of this challenge is provided by olympicin A, a plant-derived acylphloroglucinol from *Hypericum olympicum* that inhibits the NorA efflux pump in *S. aureus* and increases intracellular accumulation of fluoroquinolones. Despite its promising EPI activity, olympicin A is completely inactivated in the presence of blood, precluding systemic use, and its moderate cytotoxicity against human cell lines (IC50 of 8.9–12.5 μM) further highlights the translational hurdles faced by many phytochemical adjuvants [46].

The cytotoxicity profiles of representative phytochemical adjuvants discussed in this review, along with their selectivity indices where available, are summarized in Table 8. These data illustrate the range of therapeutic windows and highlight compounds with favorable selectivity profiles that warrant further development.

Poli and Motireddy (2025) recently reviewed the toxic effects of medicinal plants in human and animal models, emphasizing the need for comprehensive toxicological assessments before plant-derived compounds can be considered for clinical use [145].

For instance, ajoene, despite its robust QS-inhibitory and antibiofilm activities at concentrations of 80–100 μg/mL, induces apoptosis in human A549 lung epithelial cells with an EC50 of approximately 100 μM (23 μg/mL) and inhibits cell proliferation with an IC50 of approximately 100 μM [90]. Although this cytotoxicity profile was approximately 10-fold more favorable than that of the synthetic furanone C-30, it underscores the challenge of achieving a therapeutic window sufficient for systemic administration, particularly for chronic infections requiring prolonged treatment [90].

The selectivity of phytochemical adjuvants for bacterial over mammalian cells is influenced by several factors, including differences in membrane lipid composition, the presence or absence of specific target proteins, and the differential expression of drug-metabolizing enzymes [53,105]. Exploiting these differences to design or select compounds with improved selectivity is a key goal of medicinal chemistry optimization efforts. Structure–activity relationship (SAR) studies, such as those conducted by Omosa et al. (2016) [51] on compounds from Kenyan plants and by Michalet et al. (2007) [50] on N-caffeoylphenalkylamide derivatives, can guide the identification of chemical modifications that enhance antibacterial adjuvant activity while reducing mammalian cytotoxicity.

The translational gap created by unresolved toxicity concerns can only be bridged through systematic and rigorous toxicological evaluation. This should include in vitro cytotoxicity assays against relevant human cell lines, assessment of hemolytic activity, and in vivo toxicity studies in animal models, following standardized protocols and guidelines [33,54]. Furthermore, the potential for phytochemical adjuvants to interact with the pharmacokinetics of co-administered antibiotics, either by inhibiting or inducing drug-metabolizing enzymes or transporters, must be carefully evaluated to avoid unintended changes in antibiotic exposure that could lead to therapeutic failure or increased toxicity [22,29].

From an evolutionary perspective, the selection of resistance mechanisms targeted by phytochemical adjuvants carries important implications for the long-term sustainability of these strategies. Efflux pumps, quorum sensing systems, and biofilm-forming pathways are evolutionarily ancient and highly conserved across bacterial lineages, suggesting that they confer significant fitness advantages under natural environmental conditions [146]. The inhibition of these mechanisms by phytochemical adjuvants may therefore impose a fitness cost on bacteria, potentially reducing their competitive ability in polymicrobial communities and limiting the spread of resistance [86,146]. However, bacteria have demonstrated a remarkable capacity to evolve compensatory mutations that mitigate fitness costs associated with resistance, and it is possible that prolonged exposure to phytochemical adjuvants could select for strains that no longer rely on the targeted mechanism for survival, or that evolve alternative resistance pathways [147]. For instance, chronic inhibition of quorum sensing could theoretically select for “cheater” mutants that no longer produce QS signals but still benefit from the public goods produced by wild-type populations, or for strains that activate virulence pathways through QS-independent mechanisms [86]. Incorporating evolutionary biology principles into the design and evaluation of phytochemical adjuvants will be essential to anticipate and mitigate these risks. Strategies such as combining adjuvants with different mechanisms of action, rotating adjuvant-antibiotic combinations, and targeting mechanisms that are tightly linked to essential cellular functions may help to minimize the evolutionary escape potential of bacterial pathogens [146,147].

### 4.4. Overcoming the Gap: Nanotechnology and Advanced Delivery Systems

Nanotechnology has emerged as a transformative approach to address the pharmacokinetic and toxicity limitations of phytochemical adjuvants [34,118]. By encapsulating plant-derived compounds within nanoscale delivery vehicles, it is possible to enhance their aqueous solubility, protect them from metabolic degradation, prolong their circulation time, and achieve targeted delivery to the site of infection [114,119]. These advantages can collectively increase the therapeutic index of phytochemical adjuvants, bringing effective in vivo concentrations within a clinically achievable range.

A variety of nanocarrier platforms have been investigated for the delivery of plant-derived antimicrobials and adjuvants. Liposomes, which are spherical vesicles composed of phospholipid bilayers, can encapsulate both hydrophilic and hydrophobic phytochemicals and have been shown to improve the delivery of essential oils and flavonoids to bacterial biofilms [114,148]. Polymeric nanoparticles, prepared from biodegradable polymers such as poly(lactic-co-glycolic acid) (PLGA), offer controlled release properties and can be surface-functionalized with targeting ligands that recognize bacterial surface structures or biofilm matrix components [58,118]. Nanoemulsions and solid lipid nanoparticles have also been employed to improve the solubility and stability of hydrophobic terpenes and essential oils, enhancing their antibacterial and antibiofilm activity [48,75].

The application of nanotechnology to phytochemical adjuvants is not limited to improving pharmacokinetics; it can also enable combination therapy at the nanoscale. Co-encapsulation of a phytochemical adjuvant and a conventional antibiotic within the same nanocarrier can ensure that both agents reach the site of infection simultaneously and at the optimal ratio for synergy, an approach that has shown promise in preclinical studies [107]. Furthermore, stimuli-responsive nanocarriers that release their payload in response to specific triggers present at the infection site, such as low pH, bacterial enzymes, or reactive oxygen species, can further enhance the selectivity and efficacy of phytochemical–antibiotic combinations [114,116].

An advanced strategy to enhance the therapeutic index of phytochemical-loaded nanocarriers involves their surface functionalization with targeting ligands that recognize specific bacterial surface structures, thereby achieving selective delivery to pathogenic bacteria while minimizing off-target effects on host cells and commensal microbiota. Several targeting approaches have been explored with promising results. Liposomes and nanoparticles have been developed for the targeted delivery of antibiotics to bacterial infection sites, with surface functionalization enabling preferential accumulation at the site of infection and enhanced antibacterial efficacy [149]. Aptamers, short single-stranded oligonucleotides selected for high-affinity binding to bacterial surface proteins, represent a particularly promising targeting strategy. Ommen et al. (2022) demonstrated that aptamer-functionalized nanoparticles achieve specific targeting and enhanced drug delivery to *Staphylococcus aureus* biofilms, significantly improving antibiofilm efficacy compared to non-targeted formulations [150]. A comprehensive review by Ye et al. (2024) further highlighted the versatility of aptamer-based strategies in combating bacterial infections, spanning applications from diagnostics to targeted therapeutics, and discussed the potential of aptamers to direct antimicrobial agents, including phytochemicals, specifically to pathogenic bacteria while sparing commensal flora [151]. While the application of these advanced targeting strategies to plant-derived antibiotic adjuvants is still in its early stages, the proof-of-concept demonstrated with conventional antibiotics and the growing interest in phytochemical nanoformulations suggest that targeted delivery systems could significantly improve the therapeutic index and clinical applicability of plant-derived adjuvants in the near future.

Despite the considerable promise of nanotechnology-based approaches, several challenges remain for their clinical translation. These include the scalability and reproducibility of nanocarrier manufacturing, the potential immunogenicity and long-term toxicity of nanocarrier materials, and the regulatory complexities associated with the approval of nanomedicine products [34,119]. Nevertheless, the rapid pace of innovation in this field, coupled with the growing urgency of the AMR crisis, provides strong impetus for continued research and development in this area.

### 4.5. Bridging the Gap: From In Vitro to In Vivo Models

The translation of promising in vitro findings to clinically relevant outcomes requires rigorous evaluation in appropriate in vivo models that recapitulate key features of human ESKAPE infections [108,110]. The scarcity of such studies represents one of the most significant gaps in the current literature on plant-derived antibiotic adjuvants, as the vast majority of published research remains confined to in vitro assays [29,30].

Invertebrate models, particularly the greater wax moth *Galleria mellonella*, have gained popularity as an intermediate step between in vitro assays and mammalian models [128,152]. *G. mellonella* larvae possess an innate immune system with remarkable functional similarities to that of mammals, including phagocytic hemocytes and the production of antimicrobial peptides, and they can be maintained at 37 °C, the physiological temperature for human pathogens [128]. The model has been successfully employed to evaluate the in vivo efficacy of antimicrobial agents against a range of ESKAPE pathogens, including *Enterobacter cloacae* [111], and to assess the virulence of bacterial strains and their response to treatment [152]. As a concrete example, berberine, a plant alkaloid with broad-spectrum antibacterial activity, was evaluated in a *G. mellonella* infection model against uropathogenic *E. coli*, where it significantly increased larval survival and reduced bacterial load in the hemolymph, providing proof-of-concept for the in vivo efficacy of a phytochemical against a clinically relevant pathogen [30]. Similarly, the nematode *Caenorhabditis elegans* and the brine shrimp *Artemia* spp. nauplii have been employed as invertebrate models to validate the anti-infective efficacy of plant-derived quorum sensing inhibitors. Angusamy et al. (2023) demonstrated that phytochemicals such as rosmarinic acid, naringin, and morin significantly increased the survival of *Artemia* spp. nauplii infected with *Vibrio* spp. and rescued *C. elegans* from *P. aeruginosa* PAO1 and clinical isolate infection, without exhibiting toxicity at the tested concentrations. These findings provide important proof-of-concept that plant-derived QS inhibitors can exert anti-infective effects in live animal models [89].

In addition to the invertebrate models discussed above, the zebrafish (*Danio rerio*) embryo and larva model has emerged as a valuable intermediate platform that bridges the gap between invertebrate and mammalian infection models. Zebrafish embryos are optically transparent, allowing for real-time visualization of fluorescently labeled bacteria and host immune responses at high resolution, and they possess both innate and adaptive immune components that share significant homology with those of mammals [131]. Importantly, zebrafish can be maintained at 28–37 °C, enabling the study of clinically relevant human pathogens, and their small size and high fecundity make them amenable to medium-throughput screening. The model has been successfully employed to evaluate the in vivo efficacy and toxicity of antimicrobial agents, including antibiotics, against a range of bacterial pathogens [131]. Jijie et al. (2021) reviewed the utility of zebrafish as a screening model to study the single and joint effects of antibiotics, highlighting its value for assessing both efficacy and toxicity of antimicrobial compounds in a whole-organism context [153]. Furthermore, Rasheed et al. (2021) demonstrated that zebrafish is an attractive model to study *Staphylococcus aureus* infection and can be used as a drug discovery tool, validating its applicability to ESKAPE-relevant pathogens [154]. Despite these promising examples, the use of zebrafish models specifically for the evaluation of plant-derived antibiotic adjuvants remains limited, representing a significant opportunity for future translational research. The zebrafish model offers a unique combination of biological complexity, experimental tractability, and ethical acceptability that positions it as an ideal intermediate step between invertebrate and murine models in the translational development of phytochemical adjuvants.

The low cost, ethical acceptability, and ease of handling of *G. mellonella* make it an attractive screening tool for prioritizing phytochemical–antibiotic combinations for further evaluation in mammalian models [126].

Mammalian models, particularly murine models of infection, remain the gold standard for preclinical evaluation of anti-infective agents and are essential for generating the pharmacokinetic, pharmacodynamic, and toxicological data required for regulatory approval [126,127]. However, the number of studies that have evaluated plant-derived antibiotic adjuvants in murine models of ESKAPE infections is remarkably limited. The few available studies have focused primarily on the evaluation of essential oils and their components, often using topical or intranasal administration routes that bypass the systemic bioavailability challenges discussed in Section 4.1 [75,155]. Furthermore, the evaluation of plant-derived phytocompounds in veterinary models, such as the use of *Punica granatum* against gastrointestinal nematodes in sheep, provides additional evidence for the in vivo efficacy of plant-based therapies, although their relevance to human ESKAPE infections remains to be established [156]. Encouragingly, the in vivo efficacy of ajoene has been demonstrated in a murine pulmonary infection model, where prophylactic and post-infection treatment with ajoene at 25 μg/g body weight resulted in a more than 500-fold reduction in *P. aeruginosa* bacterial load compared to untreated controls by day 3 post-infection [90]. This significant clearance, comparable to that achieved with a lasR rhlR double mutant, provides proof-of-concept that QS inhibition alone can produce clinically meaningful outcomes in vivo [90]. The development of appropriate murine models that replicate the clinical scenarios in which antibiotic adjuvants are most needed, including biofilm-associated chronic wound infections, ventilator-associated pneumonia, and catheter-related bloodstream infections, is a critical priority for advancing the field [56,126]. In parallel, the refinement of in vitro biofilm models, such as the Calgary Biofilm Device, the Biofilm Ring Test, and microfluidic systems like the BiofilmChip, can provide more physiologically relevant platforms for the initial screening of antibiofilm phytochemicals [56]. Furthermore, the application of machine learning and computational approaches, including molecular docking and QSAR modeling, holds considerable promise for accelerating the discovery and optimization of plant-derived antibiofilm agents, as recently demonstrated by ML-based virtual screening efforts that achieved hit rates up to 26% for FDA-approved compounds [56].

The principal invertebrate and mammalian models that have been employed to validate the in vivo efficacy of plant-derived antibiotic adjuvants against ESKAPE pathogens are summarized in Table 9. These models represent critical intermediate steps in the translational pipeline, bridging the gap between promising in vitro findings and potential clinical applications.

An additional consideration that warrants greater attention in the development of antibiotic adjuvants is their potential impact on the host microbiome. Broad-spectrum antibiotics are well known to cause collateral damage to commensal bacterial communities, leading to dysbiosis that can predispose patients to secondary infections such as *Clostridioides difficile* colitis and can facilitate the colonization and expansion of resistant pathogens [134]. Theoretically, antibiotic adjuvants that specifically target resistance mechanisms or virulence factors in ESKAPE pathogens without exerting direct bactericidal activity could spare the commensal microbiota, representing a significant advantage over conventional broad-spectrum antibiotics [135]. However, this theoretical benefit remains largely unverified experimentally. Certain plant-derived compounds, particularly membrane-active agents such as essential oil components and antimicrobial peptides, may exhibit broad-spectrum activity that extends beyond the target pathogen, potentially disrupting beneficial microbial communities [74,135]. Conversely, highly specific adjuvants, such as quorum sensing inhibitors that target species-specific signaling pathways, may offer a more microbiome-sparing approach, although their spectrum of activity must be carefully characterized [86]. Future preclinical and clinical studies should therefore incorporate microbiome analysis, using metagenomic sequencing and metabolomic profiling, to assess the impact of phytochemical adjuvants on host-associated microbial communities and to identify strategies that maximize pathogen-selective activity while minimizing collateral damage to the commensal flora [134,135].

Future translational research in this field should prioritize a stepwise approach that begins with rigorous in vitro characterization of synergy and mechanism of action, progresses through evaluation in *G. mellonella* or other invertebrate models for initial in vivo validation, and culminates in comprehensive pharmacokinetic, pharmacodynamic, and efficacy studies in relevant mammalian infection models [126,128]. This approach, combined with parallel efforts to optimize the formulation and delivery of phytochemical adjuvants through nanotechnology and medicinal chemistry, will be essential to bridge the translational gap and realize the clinical potential of plant-derived antibiotic adjuvants against drug-resistant ESKAPE pathogens. Collectively, addressing these translational hurdles requires a coordinated, multidisciplinary effort that integrates natural product chemistry, medicinal chemistry, nanotechnology, and rigorous in vivo validation.

An emerging and potentially transformative concept is the integration of plant-derived antibiotic adjuvants with rapid diagnostic tests, some of which may themselves be derived from plant-based components. The rational deployment of antibiotic adjuvants requires precise knowledge of the resistance mechanisms present in the infecting pathogen, as an efflux pump inhibitor, for example, would be ineffective against a strain whose resistance is mediated exclusively by enzymatic inactivation [18,157]. Recent advances in rapid diagnostics, including CRISPR-based detection systems, lateral flow immunoassays, and colorimetric sensors, many of which utilize plant-derived enzymes, lectins, or chromogenic substrates, now enable the identification of specific resistance determinants within hours rather than days [157,158]. The pairing of these diagnostics with resistance mechanism-specific phytochemical adjuvants could enable a precision medicine approach to infectious diseases, wherein the appropriate adjuvant-antibiotic combination is selected based on the resistance profile of the infecting pathogen. For instance, a rapid diagnostic test confirming NorA-mediated fluoroquinolone resistance in an MRSA isolate could guide the co-administration of a flavonoid-based efflux pump inhibitor with ciprofloxacin, while a negative result would spare the patient unnecessary exposure to the adjuvant [15]. This diagnostic-adjuvant paradigm, while still in its infancy, represents a promising avenue to maximize therapeutic efficacy while minimizing off-target effects and selective pressure for resistance [157].

## 5. Conclusions

The growing threat of antimicrobial resistance among ESKAPE pathogens demands innovative therapeutic strategies that extend beyond the conventional paradigm of discovering new bactericidal antibiotics. This review has critically evaluated the potential of plant-derived compounds to function as antibiotic adjuvants, restoring the efficacy of existing antibiotics through the inhibition of efflux pumps, the disruption of biofilms, the interference with quorum sensing systems, and the permeabilization of bacterial membranes. The substantial body of in vitro evidence reviewed herein demonstrates that numerous phytochemicals, including flavonoids, terpenes, organosulfur compounds, and polyphenols, can effectively re-sensitize multidrug-resistant ESKAPE pathogens to first-line antibiotics, often achieving synergistic interactions as quantified by FICI values and confirmed by time-kill kinetics.

Despite the breadth and depth of in vitro data supporting the adjuvant potential of plant-derived compounds, the translational gap between laboratory findings and clinical applications remains the most significant obstacle to their therapeutic development. The pharmacokinetic limitations of many phytochemicals, including poor aqueous solubility, extensive metabolism, and rapid elimination, result in in vivo concentrations that are often insufficient to recapitulate the adjuvant effects observed in vitro. Furthermore, concerns regarding cytotoxicity, the lack of standardized extracts, and the scarcity of robust in vivo efficacy studies in relevant animal models of ESKAPE infections have hindered the progression of even the most promising phytochemical adjuvants toward clinical evaluation.

Addressing these translational challenges will require a concerted and multidisciplinary effort spanning the fields of natural product chemistry, medicinal chemistry, pharmacokinetics, nanotechnology, and infectious disease research. The application of nanotechnology-based delivery systems, including liposomes, polymeric nanoparticles, and nanoemulsions, offers a particularly promising avenue to overcome the bioavailability and targeting limitations of phytochemical adjuvants. The co-encapsulation of phytochemical adjuvants with conventional antibiotics within these nanocarriers may further enable the simultaneous delivery of synergistic combinations at optimal ratios to the site of infection.

Finally, it is important to contextualize the potential role of plant-derived antibiotic adjuvants within the broader landscape of antimicrobial therapy. While these compounds offer a promising strategy to extend the clinical utility of existing antibiotics, they are not intended to replace conventional antibiotics, nor do they address all the limitations of broad-spectrum therapy. The ideal antibiotic adjuvant would selectively target resistance or virulence mechanisms in pathogenic bacteria while sparing the commensal microbiota, thereby reducing the collateral damage associated with broad-spectrum antibiotic treatment. However, achieving this degree of selectivity remains a formidable challenge, and many phytochemical adjuvants currently under investigation, particularly membrane-active compounds, may themselves possess broad-spectrum activity that could disrupt beneficial microbial communities. Furthermore, the deployment of antibiotic adjuvants must be integrated into comprehensive antimicrobial stewardship programs to prevent the misuse that could accelerate the emergence of resistance to both the adjuvant and the companion antibiotic. Looking forward, the most impactful contributions of plant-derived adjuvants may lie not in their use as universal potentiators of broad-spectrum antibiotics, but rather in the context of precision antimicrobial therapy, guided by rapid diagnostics and tailored to the specific resistance mechanisms present in the infecting pathogen. This vision, though aspirational, aligns with the broader shift toward personalized medicine and offers a sustainable framework for combating the AMR crisis while preserving the efficacy of both existing and future antimicrobial agents.

Equally important is the need for a standardized translational pipeline that systematically evaluates phytochemical adjuvants from in vitro synergy assays through invertebrate models such as *Galleria mellonella* and, ultimately, to rigorous efficacy and safety studies in mammalian models of clinically relevant ESKAPE infections. The adoption of standardized methodologies for evaluating synergy, characterizing mechanisms of action, and assessing pharmacokinetic and toxicological parameters will be essential to ensure the reproducibility and comparability of results across studies.

The concept of antivirulence therapy, exemplified by quorum sensing inhibitors that disarm rather than kill bacteria, represents a paradigm shift in the approach to infectious disease treatment and aligns closely with the historical role of plants as sources of anti-infective agents. By targeting non-essential virulence and resistance mechanisms, phytochemical adjuvants may impose reduced selective pressure for resistance development, potentially offering a more sustainable strategy for antimicrobial therapy.

In conclusion, plant-derived antibiotic adjuvants represent a promising and underexploited resource in the fight against drug-resistant ESKAPE pathogens. The wealth of ethnopharmacological knowledge, the chemical diversity of plant secondary metabolites, and the emerging technologies for improving their delivery and efficacy collectively provide a strong foundation for future research. Realizing the clinical potential of these compounds will require a shift in research focus from descriptive in vitro studies toward mechanism-driven translational investigations that address the pharmacokinetic, toxicological, and regulatory challenges inherent to the development of plant-derived therapeutics. The urgency of the AMR crisis, projected to cause tens of millions of deaths by 2050, demands that no promising avenue for therapeutic intervention remains unexplored.

## Figures and Tables

**Table 1:** Structural Characteristics and Resistance Features of ESKAPE Pathogens.

Pathogen	Gram Stain	Key Structural Features	Key Resistance Mechanisms	Clinical Challenge	References
*Enterococcus faecium*	Positive	Thick peptidoglycan layer; lipoteichoic acids	*van* gene cluster (vancomycin resistance); aminoglycoside-modifying enzymes	VRE (vancomycin-resistant enterococci)	[10,11]
*Staphylococcus aureus*	Positive	Thick peptidoglycan layer; protein A; capsule	*mecA* gene (PBP2a); NorA efflux pump; β-lactamase production	MRSA; biofilm-associated infections	[4,15]
*Klebsiella pneumoniae*	Negative	Outer membrane (LPS); polysaccharide capsule; thin peptidoglycan	ESBLs (CTX-M, SHV, TEM); carbapenemases (KPC, NDM, OXA-48); AcrAB-TolC efflux pump	Carbapenem-resistant *K. pneumoniae* (CRKP)	[4,14]
*Acinetobacter baumannii*	Negative	Outer membrane (LPS); thin peptidoglycan; capsule	Carbapenemases (OXA-23, NDM); AdeABC efflux pump; outer membrane impermeability	XDR strains; desiccation resistance on surfaces	[4,13]
*Pseudomonas aeruginosa*	Negative	Outer membrane (LPS); thin peptidoglycan; low membrane permeability (12–100× less than *E. coli*)	MexAB-OprM, MexXY-OprM efflux pumps; AmpC β-lactamase; biofilm formation	Intrinsic multidrug resistance; biofilm tolerance	[12,13]
*Enterobacter* species	Negative	Outer membrane (LPS); thin peptidoglycan	AmpC β-lactamase (inducible); ESBLs; carbapenemases	Chromosomal AmpC derepression during therapy	[4,14]

The table summarizes the Gram staining classification, key structural features, major resistance mechanisms, clinical challenges, and references for each ESKAPE pathogen. LPS: lipopolysaccharide; ESBL: extended-spectrum β-lactamase; KPC: Klebsiella pneumoniae carbapenemase; NDM: New Delhi metallo-β-lactamase; OXA: oxacillinase; MRSA: methicillin-resistant Staphylococcus aureus; VRE: vancomycin-resistant enterococci; XDR: extensively drug-resistant.

**Table 2:** Plant-Derived Efflux Pump Inhibitors (EPIs) and Their Targets in ESKAPE Pathogens.

Phytochemical/ Extract	Plant Source	Efflux Pump Family/Pump Targeted	Target ESKAPE Pathogen (s)	References
Quercetin	Various fruits and vegetables	MFS (NorA)	*S. aureus*	[15]
Kaempferol	Various fruits and vegetables	MFS (NorA)	*S. aureus*	[15]
Apigenin	Various fruits and vegetables	MFS (NorA)	*S. aureus*	[15]
Olympicin A	*Hypericum olympicum*	MFS (NorA)	*S. aureus*	[46]
Carnosic acid/Carnosol	*Rosmarinus officinalis*	MFS	*S. aureus*	[47]
Carvacrol/Thymol	*Thymus vulgaris*, *Origanum vulgare*	RND (MexAB-OprM, AcrAB-TolC)	*P. aeruginosa*, *K. pneumoniae*, *A. baumannii*	[48]
α-Pinene	Various essential oils	RND	*A. baumannii*, *K. pneumoniae*	[48]
Plumbagin	*Plumbago* spp.	Substrate of RND efflux pumps (potentiated by PAβN)	MDR *E. coli*, *E. aerogenes*, *K. pneumoniae*, *P. aeruginosa*	[49]
N-caffeoylphenalkylamides	*Polygonum hydropiper*, *Solanum melongena*, *Capsicum annuum*	Transcriptional downregulation of efflux genes	*S. aureus* (NorA-overexpressing strains)	[50]
Compounds from Kenyan plants (panel of 48)	Various Kenyan medicinal plants	Efflux-mediated MDR phenotype	MDR *S. aureus*, *E. coli*, *K. pneumoniae*, *P. aeruginosa*	[51]

The table summarizes the phytochemical or plant extract, the botanical source, the efflux pump family or specific pump targeted, the affected ESKAPE pathogen(s), and the corresponding references. RND: Resistance-Nodulation-Division; MFS: Major Facilitator Superfamily; MATE: Multidrug and Toxic Compound Extrusion.

**Table 3:** Plant-Derived Compounds with Anti-Biofilm Activity Against ESKAPE Pathogens.

Phytochemical/Extract	Plant Source	Target ESKAPE Pathogen (s)	Mechanism of Action	References
Epigallocatechin gallate (EGCG)	*Camellia sinensis* (green tea)	*S. aureus*, *P. aeruginosa*	Inhibition of cell adhesion; QS suppression; EPS disruption	[67,68]
Resveratrol	Grapes, red wine	MRSA	Reduction of PIA, eDNA release, and ROS production	[69]
Quercetin	Various fruits and vegetables	*P. aeruginosa*, *S. aureus*	Biofilm inhibition and dispersal	[66,67]
*Alpinia nigra* leaf extract (ethyl acetate)	*Alpinia nigra*	*P. aeruginosa* PAO1	QS inhibition; EPS reduction; downregulation of *lasI*, *lasR*, *lasB*, *rhlI*, *rhlR*, *rhlA*	[72]
*Matayba oppositifolia* leaf extract (methanolic)	*Matayba oppositifolia*	MRSA, MDRSA	Biofilm inhibition and eradication (IC50 = 10.4 μg/mL)	[66]
*Quercus robur* extract	*Quercus robur* (oak)	*S. aureus* (clinical isolates and MRSA ATCC 43300)	QS interference; EPS synthesis disruption; membrane permeabilization	[61]
Aloe honey (HO1)	Medicinal plant-derived honey	*S. aureus*, *Salmonella typhimurium*	Biofilm inhibition (89.26% reduction); β-lactamase inhibition	[76]
Manuka honey	*Leptospermum scoparium*	MRSA, *P. aeruginosa*	Biofilm architecture disruption; QS inhibition; antibiotic susceptibility restoration	[77,78,79,80]

The table summarizes the phytochemical or extract, the botanical source, the target ESKAPE pathogen(s), the proposed mechanism of anti-biofilm action, and the corresponding references. IC50: half-maximal inhibitory concentration; EPS: extracellular polymeric substance; QS: quorum sensing; PIA: polysaccharide intercellular adhesin; eDNA: extracellular DNA; ROS: reactive oxygen species.

**Table 4:** Plant-Derived Quorum Sensing Inhibitors and Their Antivirulence Effects on ESKAPE Pathogens.

Phytochemical/ Extract	Plant Source (as Reported)	QS System Targeted	Antivirulence Effect (s)	Target Pathogen (s)	References
Ajoene	*Allium sativum*	las, rhl	Reduced rhamnolipid & elastase production; Biofilm sensitization	*P. aeruginosa*	[90]
Curcumin	*Curcuma longa*	AHL-dependent	Biofilm inhibition, Reduced virulence factor production	*P. aeruginosa*	[92]
Quercetin	Various fruits/vegetables	AHL-dependent	Biofilm inhibition and dispersal	*P. aeruginosa*	[70]
*Alpinia nigra* leaf extract	*Alpinia nigra*	AHL-dependent	Suppression of QS-regulated virulence factors, Biofilm inhibition	*P. aeruginosa* PAO1	[72]
Vanillin	*Vanilla planifolia*	AHL-dependent	Cecal microbiome modulation, Reduced inflammation (in vivo)	Gut microbiome (Enterobacteriaceae, *Salmonella* spp.) in broiler chicken model	[97]
Umbelliferone	Apiaceae family	AHL-dependent	Cecal microbiome modulation, Reduced inflammation (in vivo)	Gut microbiome (Enterobacteriaceae, *Salmonella* spp.) in broiler chicken model	[97]

The table details the phytochemical or plant extract, the botanical source, the QS system or regulatory pathway targeted, the specific antivirulence phenotypes attenuated, the target ESKAPE pathogen(s), and the corresponding references. AHL: N-acyl homoserine lactone; AIP: autoinducing peptide.

**Table 5:** Plant-Derived Compounds with Membrane-Permeabilizing Activity Against ESKAPE Pathogens.

Phytochemical/Extract	Plant Source	Target ESKAPE Pathogen (s)	Mechanism of Action	References
Terpinen-4-ol	*Melaleuca alternifolia* (tea tree oil)	*S. aureus*, *P. aeruginosa*	Membrane integrity disruption; leakage of intracellular ions; potentiation of aminoglycosides and beta-lactams	[108]
Nerol	*Cymbopogon citratus* (lemongrass) and other essential oils	MDR Gram-negative bacteria	Membrane disruption; synergistic effects with fluoroquinolones	[109]
Eugenol	*Syzygium aromaticum* (clove oil)	Gram-negative bacteria	Outer membrane permeabilization; enhanced intracellular accumulation of ampicillin and erythromycin	[110,111]
Baicalin	*Scutellaria baicalensis* (Chinese skullcap)	MDR pathogens	Membrane disruption; biofilm inhibition; efflux pump inhibition (multi-mechanistic)	[106,112]
Carvacrol/Thymol	*Thymus vulgaris*, *Origanum vulgare*	*A. baumannii*, *P. aeruginosa*, *K. pneumoniae*	Outer membrane permeabilization; disruption of lipid packing	[50,75]
*Matayba oppositifolia* extract (n-hexane bark)	*Matayba oppositifolia*	Carbapenem-resistant *K. pneumoniae*, XDR *A. baumannii*	Membrane disruption by fatty acids, terpenes, and steroids	[117]

The table summarizes the phytochemical or extract, the botanical source, the target ESKAPE pathogen(s), the proposed mechanism of membrane-permeabilizing action, and the corresponding references. MDR: multidrug-resistant; XDR: extensively drug-resistant.

**Table 6:** Representative plant-derived compounds and extracts with demonstrated or potential antibiotic adjuvant activity against ESKAPE pathogens.

Phytochemical Adjuvant	Antibiotic	Target ESKAPE Pathogen (s)	Proposed Mechanism of Synergy	Effect on Antibiotic Efficacy	References
Quercetin	Ciprofloxacin/Norfloxacin	*S. aureus* (including MRSA)	NorA efflux pump inhibition	Significant MIC reduction; FICI ≤ 0.5	[15]
Kaempferol	Ciprofloxacin	*S. aureus* (including MRSA)	NorA efflux pump inhibition	Significant MIC reduction; Synergistic (FICI ≤ 0.5)	[15]
Carnosic acid/ Carnosol	Erythromycin/ Tetracycline	*S. aureus*	Efflux pump inhibition (MFS)	Restoration of antibiotic susceptibility	[47]
Carvacrol/Thymol	Colistin/ Rifampicin/ Macrolides	*A. baumannii*, *P. aeruginosa*, *K. pneumoniae*	Outer membrane permeabilization	Potentiation of antibiotic entry; Synergistic killing	[48,75]
Ajoene	Tobramycin	*P. aeruginosa*	Quorum sensing inhibition (las/rhl); Biofilm sensitization	Eradication of tobramycin-refractory biofilms	[90]
*Alpinia nigra* extract	Not tested in combination (kanamycin used only as positive control)	*P. aeruginosa* PAO1	QS inhibition (*lasI*, *lasR*, *lasB*, *rhlI*, *rhlR*, *rhlA* downregulation); Biofilm suppression; EPS reduction	Direct anti-biofilm and anti-QS activity; no synergy data available	[72]
*Matayba oppositifolia* extract	Not tested in combination	Carbapenem-resistant *K. pneumoniae*, XDR *A. baumannii*	Membrane disruption	Direct antimicrobial activity; potential for future combination studies	[117]
N-caffeoylphenalkylamides	Fluoroquinolones	MDR bacteria	Efflux pump gene downregulation	Potentiation of fluoroquinolone activity	[50]

The table summarizes the phytochemical adjuvant, the co-administered antibiotic, the target pathogen(s), the mechanism(s) underlying the synergistic interaction, the reported effect on the antibiotic MIC or FICI, and the corresponding references. FICI: Fractional Inhibitory Concentration Index; MIC: Minimum Inhibitory Concentration; MRSA: Methicillin-Resistant *Staphylococcus aureus*; MDR: Multidrug-Resistant.

**Table 7:** Translational Challenges and Mitigation Strategies for Plant-Derived Antibiotic Adjuvants.

Challenge	Description	Mitigation Strategies	References
Poor bioavailability	Low aqueous solubility, extensive first-pass metabolism, rapid elimination	Nanoformulations (liposomes, SLNs, polymeric nanoparticles); bioenhancers (e.g., piperine); structural analogs	[33,34,118]
Standardization	Batch-to-batch variability; complex multi-component mixtures	Standardized extracts with defined chemical fingerprints; chromatographic/spectroscopic profiling; single active compound isolation	[29,130]
Cytotoxicity	Non-selective membrane disruption; mitochondrial toxicity	SAR-guided optimization; selective targeting ligands; sub-inhibitory concentrations for adjuvant effect	[33,49]
Limited in vivo validation	Majority of studies confined to in vitro assays	Stepwise evaluation in *G. mellonella*, zebrafish, and murine models; clinically relevant infection models	[126,128,131]
Economic barriers	IP protection challenges; small market size; low-cost supplement competition	Public–private partnerships; non-profit development models; delinked reimbursement	[132,133]
Microbiome impact	Potential collateral damage to commensal flora	Highly specific adjuvants (e.g., QS inhibitors); targeted delivery; microbiome monitoring	[134,135]
Regulatory pathway	Unclear approval framework for adjuvant-antibiotic combinations	Dialogue with regulatory agencies; establishment of guidelines for combination therapies	[17,18]

The table summarizes the major translational challenges facing the development of plant-derived antibiotic adjuvants, their descriptions, potential mitigation strategies, and key references. SLNs: solid lipid nanoparticles; SAR: structure–activity relationship; QS: quorum sensing; IP: intellectual property.

**Table 8:** Cytotoxicity Profiles of Representative Phytochemical Adjuvants Against Mammalian Cell Lines.

Phytochemical	Test System	Cytotoxicity (CC_50_ or IC_50_)	Effective Antibacterial Concentration	Selectivity Index (SI)	References
Quercetin	Human hepatocytes (HepG2)	IC_50_ = 61.5 μM	MIC = 16–64 μg/mL against *S. aureus*	>3.8 (based on MIC)	[15,33]
Ajoene	Human A549 lung epithelial cells	EC_50_ ≈ 100 μM (23 μg/mL)	80–100 μg/mL (QS inhibition)	~1 (QS inhibition); ~10-fold better than furanone C-30	[90]
Olympicin A	Human cell lines (various)	IC_50_ = 8.9–12.5 μM	MIC = 0.5–2 μg/mL against *S. aureus*	Moderate; inactivated in blood	[46]
Curcumin	Human keratinocytes (HaCaT)	IC_50_ ≈ 50 μM	MIC = 16–128 μg/mL against *S. aureus*, *P. aeruginosa*	>2–16 (varies by strain)	[93,137]
Carvacrol	Human intestinal cells (Caco-2)	IC_50_ = 150–250 μM	MIC = 64–256 μg/mL against Gram-negative bacteria	>3–10	[74,143]
Thymol	Human intestinal cells (Caco-2)	IC_50_ = 200–300 μM	MIC = 64–256 μg/mL against Gram-negative bacteria	>3–12	[74,143]
Berberine	Human hepatocytes (HepG2)	IC_50_ = 25–50 μM	MIC = 32–128 μg/mL; EPI activity at sub-MIC	Low; adjuvant use at sub-MIC recommended	[29,106]
Resveratrol	Human fibroblasts (MRC-5)	IC_50_ > 100 μM	Anti-biofilm at 6.25–50 μg/mL	>10 (anti-biofilm)	[69,144]
EGCG	Human gingival fibroblasts	IC_50_ ≈ 200 μM	Anti-biofilm at 12.5–100 μg/mL	>8 (anti-biofilm)	[67,68]

The table summarizes the cytotoxicity (CC_50_ or IC_50_) of representative phytochemical adjuvants against mammalian cell lines, their effective antibacterial or adjuvant concentrations, the calculated or estimated Selectivity Index (SI = CC_50_/effective concentration), and the corresponding references. SI values >10 are generally considered favorable for further development. QS: quorum sensing; EPI: efflux pump inhibitor; MIC: minimum inhibitory concentration.

**Table 9:** Invertebrate and Mammalian Models Used for In Vivo Validation of Plant-Derived Antibiotic Adjuvants.

Model Organism	Phytochemical/Adjuvant Tested	Target Pathogen	Main Finding	Reference
*Galleria mellonella*	Berberine	Uropathogenic *E. coli*	Increased larval survival; reduced bacterial load	[30]
*Caenorhabditis elegans*	Rosmarinic acid, naringin, morin	*P. aeruginosa* PAO1	Rescued nematodes from infection	[89]
*Artemia* spp. nauplii	Rosmarinic acid, naringin, morin	*Vibrio* spp.	Increased survival of infected nauplii	[89]
Zebrafish (Danio rerio)	Not yet applied to phytochemical adjuvants	*S. aureus*, *P. aeruginosa*	Established model for antimicrobial efficacy and toxicity screening; poised for future adjuvant studies	[131,153,154]
Murine pulmonary model	Ajoene	*P. aeruginosa*	500-fold reduction in bacterial load	[90]
Broiler chicken model	Vanillin, umbelliferone	Gut microbiome modulation	Reduced inflammation; microbiome modulation	[97]

The table summarizes the model organism employed, the phytochemical or adjuvant tested, the target pathogen, the principal in vivo finding, and the corresponding reference. These models represent critical intermediate steps in the translational pipeline for plant-derived antibiotic adjuvants.

## Data Availability

No new data were created or analyzed in this study. Data sharing is not applicable to this article.

## Abbreviations

The following abbreviations are used in this manuscript:

AHL	N-acyl homoserine lactone
AIP	Autoinducing peptide
AMR	Antimicrobial resistance
CC50	50% Cytotoxic concentration
CFU	Colony-forming unit
EC50	50% Effective concentration
EPI	Efflux pump inhibitor
EPS	Extracellular polymeric substance
ESBL	Extended-spectrum β-lactamase
ESKAPE	*Enterococcus faecium*, *Staphylococcus aureus*, *Klebsiella pneumoniae*, *Acinetobacter baumannii*, *Pseudomonas aeruginosa*, *Enterobacter* species
FIC	Fractional inhibitory concentration
FICI	Fractional inhibitory concentration index
IC50	50% Inhibitory concentration
MATE	Multidrug and toxic compound extrusion
MDR	Multidrug-resistant
MFS	Major facilitator superfamily
MIC	Minimum inhibitory concentration
MRSA	Methicillin-resistant *Staphylococcus aureus*
PLGA	Poly(lactic-co-glycolic acid)
QS	Quorum sensing
RND	Resistance-nodulation-division
SAR	Structure–activity relationship
SI	Selectivity index
WHO	World Health Organization
